# Hetero-oligomer of dynamin-related proteins participates in the fission of highly divergent mitochondria from *Entamoeba histolytica*

**DOI:** 10.1038/s41598-017-13721-5

**Published:** 2017-10-18

**Authors:** Takashi Makiuchi, Herbert J. Santos, Hiroshi Tachibana, Tomoyoshi Nozaki

**Affiliations:** 1 Department of Infectious Diseases, Tokai University School of Medicine, 143 Shimokasuya, Isehara, Kanagawa 259-1193 Japan; 2 Department of Parasitology, National Institute of Infectious Diseases, 1-23-1 Toyama, Shinjuku-ku, Tokyo, 162-8640 Japan; 3 Graduate School of Life and Environmental Sciences, University of Tsukuba, 1-1-1 Tennodai, Tsukuba, Ibaraki, 305-8572 Japan; 4 Department of Biomedical Chemistry, Graduate School of Medicine, the University of Tokyo, 7-3-1 Hongo, Bunkyo-ku, Tokyo, 113-0033 Japan

## Abstract

*Entamoeba histolytica* is an anaerobic parasitic protist and possesses mitosomes, one of the most highly divergent mitochondrion-related organelles (MROs). Although unique metabolism and protein/metabolite transport machinery have been demonstrated in *Entamoeba* mitosomes, the mechanism of mitosomal fusion and fission remains to be elucidated. In this study, we demonstrate that two dynamin-related proteins (DRPs) are cooperatively involved in the fission of *Entamoeba* mitosomes. Expression of a dominant negative form of EhDrpA and EhDrpB, and alternatively, repression of gene expression of *EhDrpA* and *EhDrpB* genes, caused elongation of mitosomes, reflecting inhibition of mitosomal fission. Moreover, EhDrpA and EhDrpB formed an unprecedented hetero-oligomeric complex with an approximate 1:2 to 1:3 ratio, suggesting that the observed elongation of mitosomes is likely caused by the disruption and instability of the complex caused by an imbalance in the two DRPs. Altogether, this is the first report of a hetero-oligomeric DRP complex which participates in the fission of mitochondria and MROs.

## Introduction

Fission and fusion of mitochondria are important to maintain the number and quality of the organelle, and are likely coordinated with their fundamental roles including the replication of mitochondrial DNA (mtDNA), the management of reactive oxygen species, and mitophagy^1^. Mitochondria, which have arisen as a consequence of an endosymbiotic event^2–5^, are clearly different from other single membrane bound organelles, e.g. the endoplasmic reticulum and endosomes, as they are segregated from the cytoplasm by double membranes, and retain mtDNA. Therefore, unlike endosomes and peroxisomes^6^, mitochondria are unable to be generated *de novo* or developed from other organelles, but must undergo elongation and fission to be segregated into daughter cells. Moreover, mitochondrial fission and fusion play an important role in the quality control of the organelle; fission allows the disposal of damaged part of mitochondria, while fusion compensates for the imbalance of mitochondrial conditions by mixing contents between normal and abnormal (or damaged) mitochondria^7,8^. Mitochondrial dynamics, particularly fission, is controlled in part by dynamin-related proteins (DRP) which belong to the dynamin GTPase superfamily^9^.

In mammalian cells, Drp1 proteins in the cytoplasm are recruited on the mitochondrial outer membrane by DRP receptors/adaptors (also known as Fis1, Mff, and Mid49/Mid51 which are single membrane spanning proteins)^10,11^ and form a homo-oligomeric spiral to coil around the mitochondrion^12^. After this process, one mitochondrion is divided into two daughter mitochondria by the constriction of the Drp1-oligomer, which is dependent on GTP hydrolysis. On the other hand, the fusion process is carried out by Mfn1/Mfn2 and Opa1, which also possess transmembrane region(s) allowing them to localize to mitochondrial outer and inner membranes, respectively^13,14^. The mitochondrial fission machinery is well conserved, in that DRPs play a central role; however, remarkable differences have been found between organisms, e.g., mammals and fungi (both of which belong to Opisthokonta), specifically in the receptors and adaptors used for recruiting DRPs on the mitochondrial outer membrane^14^.

Mitochondria have undergone remarkable changes in their compositions and functions during evolution, in particular under anaerobic or microaerophilic environment. This class of mitochondria with reduced or modified functions are called mitochondrion-related organelles (MROs) and are found in a wide range of anaerobic/microaerophilic protists and fungi^15^. In MRO-possessing protists, the proteins and molecular mechanisms for fission of MROs remain to be elucidated except for *Trichomonas vaginalis*, whose Drp (XP_001305587) was shown to be involved in the fission of hydrogenosomes^16^. *Entamoeba histolytica* is an anaerobic parasitic protist that causes dysentery and extra-intestinal abscesses and is responsible for an estimated 100,000 deaths in endemic areas annually^17^. The genus *Entamoeba* including *E*. *histolytica* possesses highly divergent MROs called mitosomes. *Entamoeba* mitosomes lack mtDNA, cristae structure, and canonical mitochondrial functions, e.g., ATP production by the tricarboxylic acid cycle and oxidative phosphorylation^18^. Instead, *Entamoeba* mitosomes have gained several unique features, not common among MROs: the sulfate activation pathway^19^, counter transport of ATP and activated sulfate (PAPS)^20^. Furthermore, *Entamoeba* mitosomes are also equipped with an outer membrane protein translocase complex containing a unique shuttle receptor (Tom60)^21^, a novel beta-barrel outer membrane protein (MBOMP30)^22^, and other lineage-specific membrane proteins^23^. Majority of these proteins are indispensable for proper cell proliferation^21,24^ and in particular, the sulfate activation pathway plays a pivotal role in stage conversion from trophozoites to cysts^25^. Despite their uniqueness and physiological importance, the mechanism of the fission of *Entamoeba* mitosomes remains totally unknown.

Here, we show that *Entamoeba* possesses genes encoding four DRP proteins as a limited panel of proteins known to be involved in mitochondrial fission in humans and yeast. We also report that two DRPs are associated with *E*. *histolytica* mitosomes, while the other two are localized in the nucleus. This distinct localization appears to be consistent with the phylogenetic inference. We further demonstrate that the expression of the GTPase-deficient mutant of the two mitosomal DRPs caused similar morphological alteration, i.e., elongation of mitosomes, suggesting that both of these DRPs are cooperatively involved in mitosomal fission. Moreover, we show that these DRPs form an unprecedented functional hetero-oligomeric complex.

## Results

### Gene survey of DRPs and other proteins known to be involved in mitochondrial dynamics

An *in silico* survey of the proteins known to be involved in mitochondrial fission and fusion indicated that none of these proteins are present in the *Entamoeba histolytica* genome, with an exception of DRPs (Supplementary Table S1). A gene survey of DRPs in the *Entamoeba* genomes was previously carried out, and two DRPs were described^26^. However, neither of the identified DRP candidates was shown to be associated with mitosomes. Therefore, we reexamined the *E*. *histolytica* genome database (AmoebaDB: http://amoebadb.org/amoeba/) to search for additional potential DRP homologs. We used the consensus sequence of the GTPase domain of the dynamin family proteins collected from various organisms from the Pfam database using the hidden Markov model (Pfam ID: PF00350) (http://pfam.xfam.org/search), as a query for our BLAST search. Aside from the two previously reported proteins, XP_649650 (therein named as EhDlp1^26^) and XP_651634^26^, we found two additional DRP candidates (XP_653348 and XP_651307). In this study, we designated these proteins (XP_649650, XP_651634, XP_653348, and XP_651307) as EhDRPs (EhDrpA, EhDrpB, EhDrpC, and EhDrpD, respectively) (Supplementary Fig. S1).

### Structure and domain prediction of DRPs and phylogenetic inference

**Figure 1:**
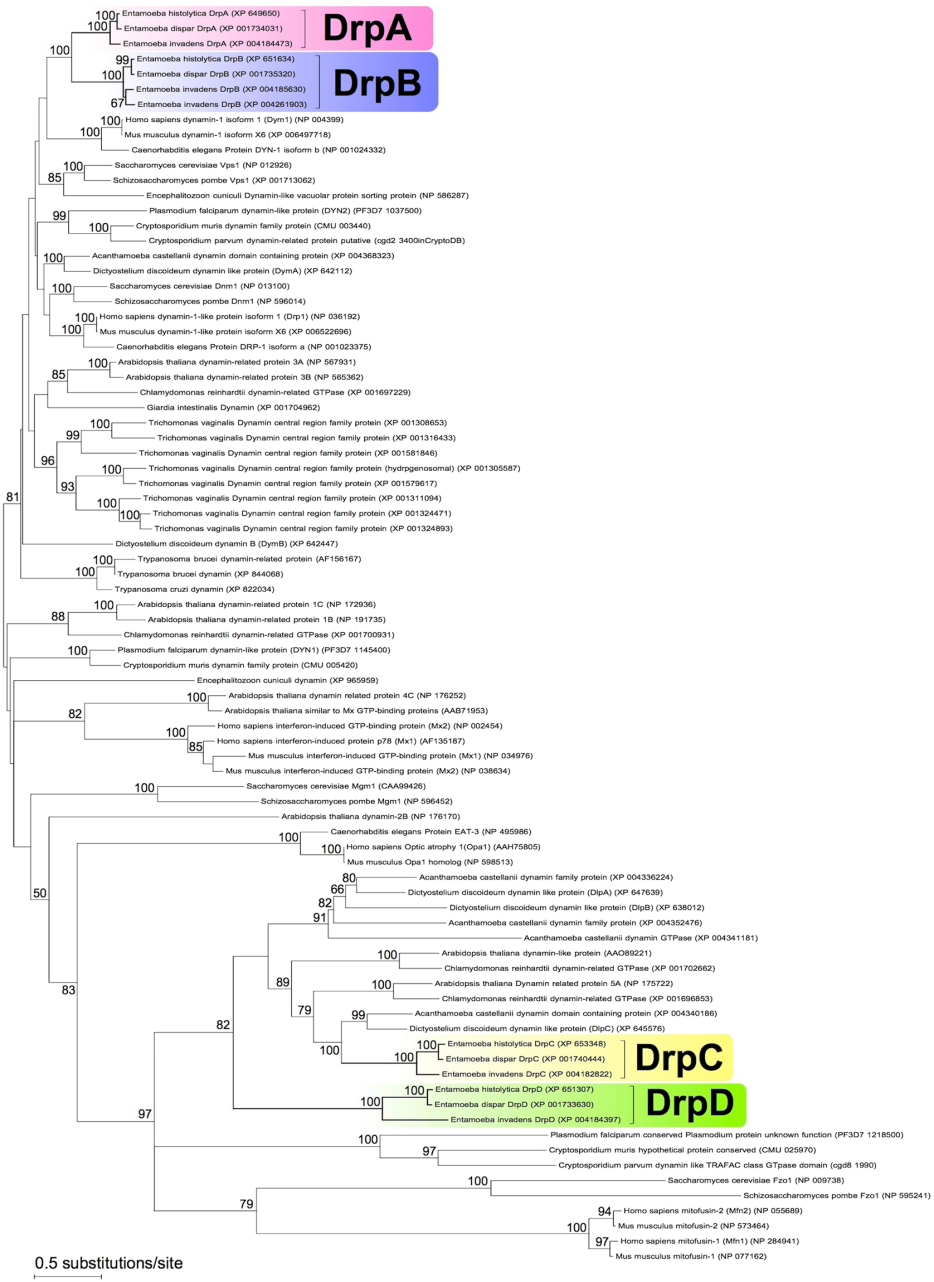
Phylogenetic reconstruction of dynamin superfamily proteins. The maximum likelihood (ML) best tree inferred by the LG model with four categories of among-site rate variation (+G, parameter = 1.4857) and the rate variation model allowed for some sites to be evolutionarily invariable (+I, 0.5029% sites). Bootstrap proportions by the ML method (100 replicates) are attached to the internal branches. Branches with less than 50% bootstrap support are unmarked.

We performed a domain search of the four EhDRPs by Pfam. EhDrpA and EhDrpB contain the GTPase domain of dynamin family (dynamin_N family, Pfam ID: PF00350), the middle domain (Dynamin_M family, Pfam ID: PF01031), and the dynamin GTPase effector domain (GED, Pfam ID: PF02212). Meanwhile both EhDrpC and EhDrpD possess only the “dynamin_N family” domain, and lack the other two domains (Supplementary Fig. S1). Therefore, these four EhDRPs seem to be classified into two groups (EhDrpA/B and EhDrpC/D) based on the composition of their predicted domains. This idea is well supported by the clustering of these EhDRPs as shown in our phylogenetic analysis (Fig. 1).

Mammalian DRPs involved in the mitochondrial fusion process, such as Opa1 and Mfn1/Mfn2, are known to contain transmembrane region(s)^13,14^. Using several predictors, we examined whether EhDRPs similarly contain transmembrane segment(s). However, no transmembrane region was predicted using TMHMM, HMMTOP, and SOSUI programs, predicting that these DRPs are not anchored to the membrane, unlike mammalian Opa1 and Mfn1/Mfn2. Meanwhile, we identified an importin α-dependent nuclear localization signal in EhDrpC by the cNLS Mapper^27^ (Supplementary Fig. S1), which confirms the observed nuclear localization (as described below).

### Cellular localization of *E*. *histolytica* DRPs

**Figure 2:**
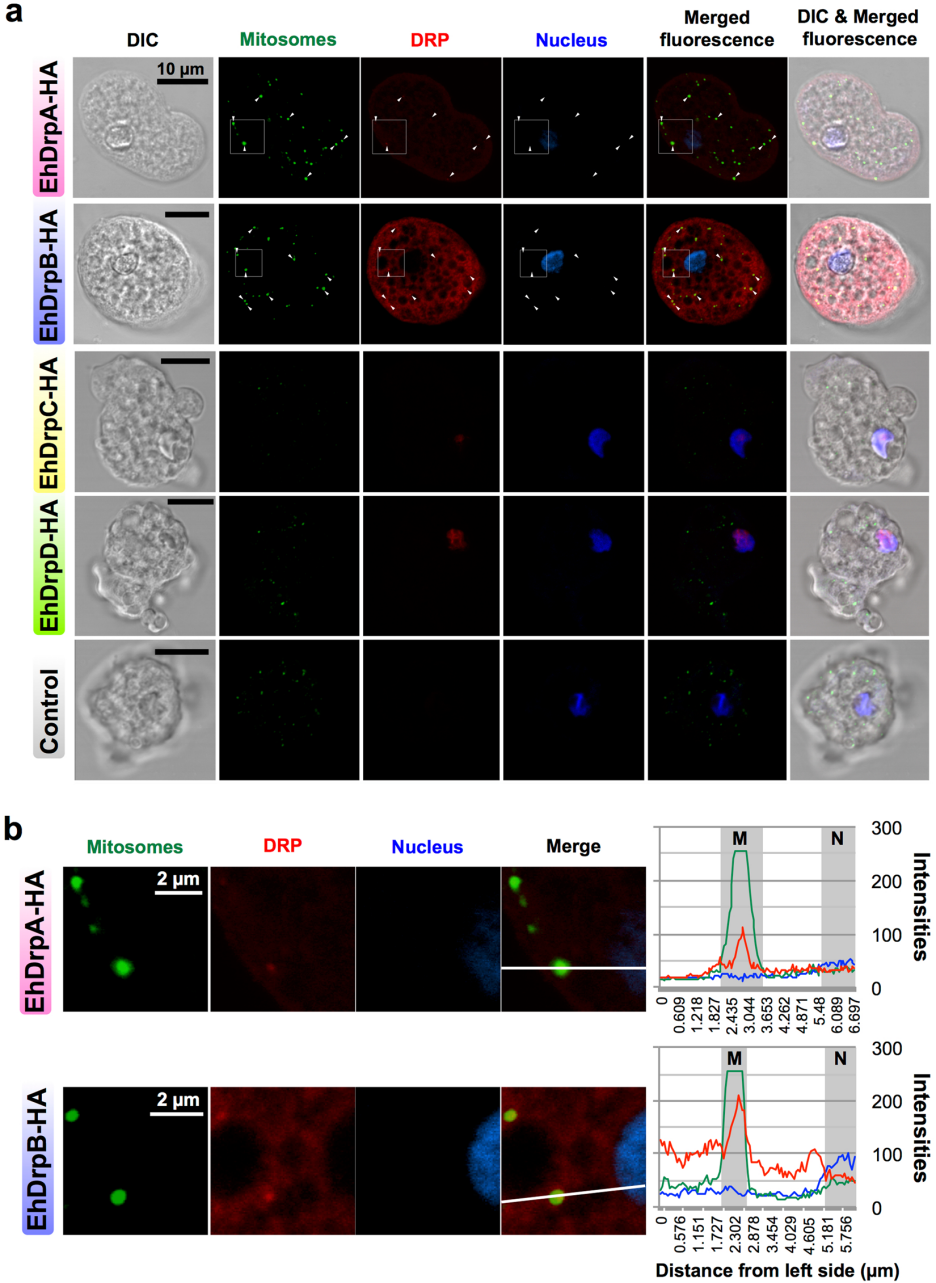
Indirect immunofluorescence analysis of the localization of *E*. *histolytica* DRPs by confocal microscopy. (**a**) Immunofluorescence images of EhDrpA-HA, EhDrpB-HA, EhDrpC-HA, and EhDrpD-HA strains. Scale bar = 10 µm. (**b**) Magnified fluorescence images of the boxed region in Fig. 1a of EhDrpA-HA and EhDrpB-HA strains. Right graphs show the profile of fluorescence intensities on the line in merged images. M and N indicate positions of mitosomes and the nucleus on the line, respectively. Scale bar = 2 µm. EhDrpA-HA, EhDrpB-HA, EhDrpC-HA, and EhDrpD-HA (red) were stained using anti-HA antibody and Alexa Fluor^®^ 594 goat anti-mouse IgG (Life Technologies). Mitosomes (green) were stained by antibody against APS kinase (APSK, mitosomal matrix protein, XP_656278) and Alexa Fluor^®^ 488 goat anti-rabbit IgG (Life Technologies). Nuclei were stained with 1 µg/ml TOTO-3 (Life Technologies) in DABCO (SIGMA). Merged images of differential interference contrast (DIC) and fluorescence are also shown. These images are approximately 1.0 µm in thickness per section. Arrowheads in Fig. 1a indicate mitosomes colocalized with the dotted signals of EhDRP-HA. Experiments were performed at least twice and more than 50 cells were observed for each strain.

As we aimed to identify a mitosome-associated DRP from *Entamoeba*, we first established *E*. *histolytica* cell lines expressing *E*. *histolytica* DRPs (EhDrpA, EhDrpB, EhDrpC, and EhDrpD) with the hemagglutinin (HA) tag at the carboxyl terminus (EhDrpA-HA, EhDrpB-HA, EhDrpC-HA, and EhDrpD-HA, respectively). After confirmation of the expression of these proteins in trophozoites by immunoblot analysis with anti-HA antibody (Supplementary Fig. S2), we performed immunofluorescence assay (IFA) using anti-HA antibody, anti-APS kinase (APSK, a mitosomal matrix marker) antibody, and TOTO-3, for staining EhDRP-HA, mitosomes^19^, and nuclei, respectively (Fig. 2a). Indirect immunofluorescence imaging showed that EhDrpA-HA and EhDrpB-HA were mainly dispersed in the cytoplasm, but occasionally colocalized with mitosomes (Fig. 2 and Supplementary Fig. S3). The distribution of the fluorescence signal across the line passing through the colocalized signals clearly indicates that the peak positions of signal intensities of EhDrpA-HA and EhDrpB-HA overlap with those of mitosomes, which was visualized with an antibody against the luminal enzyme APSK (Fig. 2b and Supplementary Fig. S3), suggesting that these EhDRPs are associated with mitosomes. Further inspection also suggested that their location was restricted to the confined area on the mitosome. The dot-like signals of EhDrpB-HA occasionally appeared to be distinctively localized to a specific peripheral part of the mitosomes (Fig. 2b). The distribution plots clearly showed that the fluorescence signal of EhDrpA-HA and EhDrpB-HA did not overlap with that of the nucleus (TOTO-3). In contrast to EhDrpA-HA and EhDrpB-HA, EhDrpC-HA and EhDrpD-HA were localized to the nucleus (Fig. 2a and Supplementary Fig. S3). To further analyze the localization of EhDRP-HA, we evaluated the Pearson correlation coefficient between intensities for EhDRP-HA and APSK or TOTO-3 from scatter diagrams generated by these intensities (Supplementary Fig. S4). The signal intensities of EhDrpA-HA and EhDrpB-HA have a weak to moderate positive correlation with that of APSK (r = 0.15 to 0.42) but have a negligible correlation with the intensity of TOTO-3 (r = −0.19 to −0.09). Meanwhile, those of EhDrpC-HA and EhDrpD-HA have a positive correlation with TOTO-3 (r = 0.22 to 0.67) but have a negligible correlation with that of APSK (r = −0.09 to 0.07). These data provides statistical support to the observation that EhDrpA/B and EhDrpC/D are associated with mitosomes and nuclei, respectively. Moreover, the distinct patterns of cellular localization of EhDrpA/B and EhDrpC/D appear to be consistent with the clustering of these EhDRPs in the phylogenetic analysis (Fig. 1), and also with the classification of the four EhDRPs based on the composition and organization of predicted domains.

### Establishment of strains expressing GTPase-deficient EhDRP-HA

To further verify whether EhDrpA and EhDrpB associate with mitosomes, we established *E*. *histolytica* cell lines expressing a mutant form of EhDrpA-HA, EhDrpB-HA, EhDrpC-HA, or EhDrpD-HA, in which the critical lysine residue for GTP hydrolysis (Supplementary Fig. S5a) was mutated to alanine, resulting in the loss of GTPase activity [EhDrpA(K38A)-HA, EhDrpB(K39A)-HA, EhDrpC(K164A)-HA, or EhDrpD(K121A)-HA, respectively]. This dominant negative strategy has been commonly used to analyze dynamins and DRPs in both unicellular and multicellular organisms^16,26,28–32^. To evaluate the effect of expression of the dominant negative GTPase-deficient EhDRPs, we created a new tetracycline (Tet)-inducible expression plasmid (pEhTex/HA, Supplementary Fig. S6) in which neomycin (G418) can be used for selection of transfectants.

We first confirmed Tet-dependent expression of a full-length protein of each HA-tagged mutant EhDRP (Supplementary Fig. S5b and c). We subsequently monitored the growth kinetics of these transformants with or without Tet induction (Supplementary Fig. S7). All four strains that express GTPase-deficient EhDRP showed either a defect or a slight retardation in growth by the addition of Tet to the medium. The level of growth defect varied among the transformant strains. The growth of the mock control strain (pEhTex/HA empty-vector) was not affected by the addition of Tet.

### Elongation of mitosomes by the expression of GTPase-deficient EhDrpA and EhDrpB

**Figure 3:**
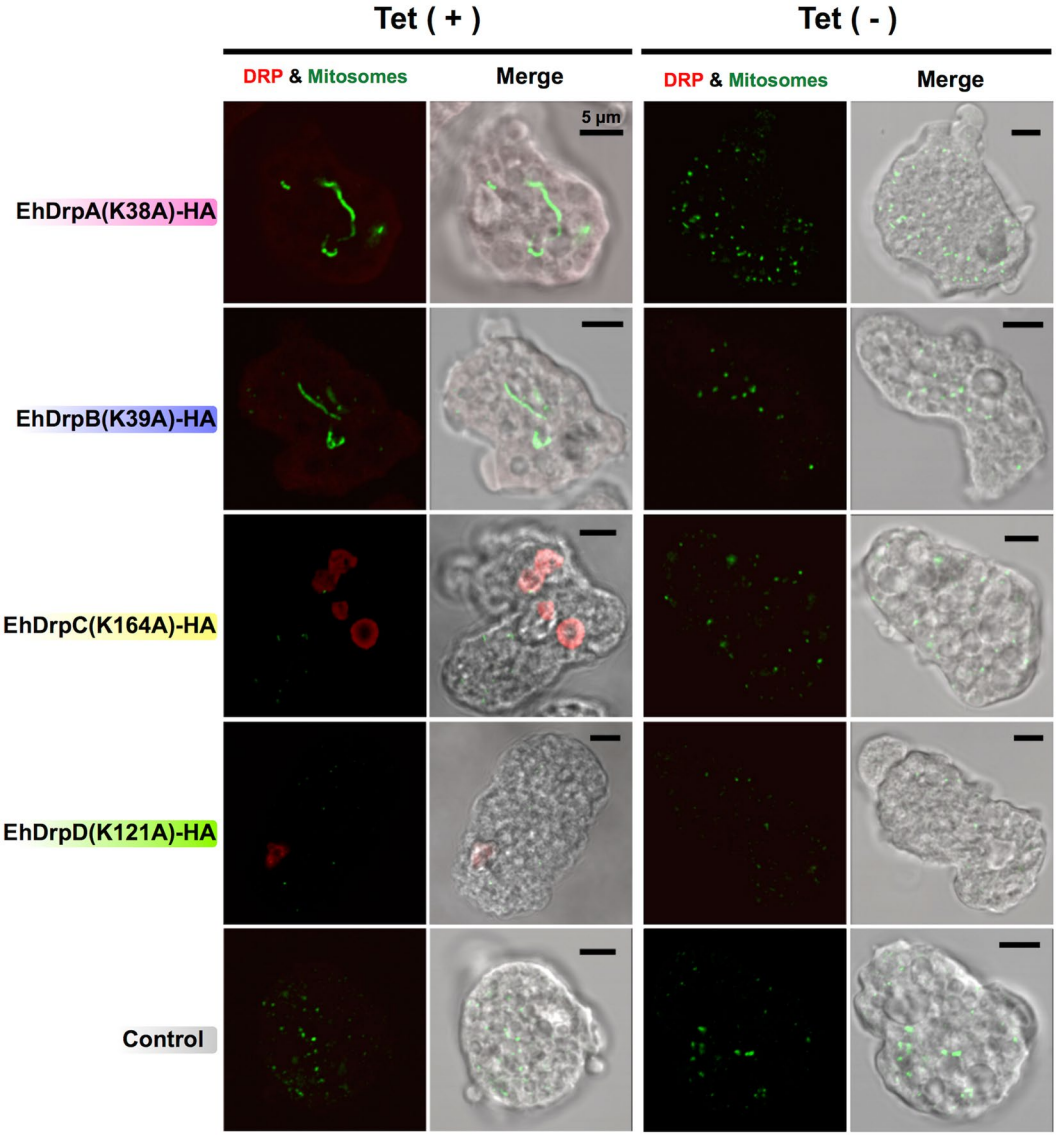
Confocal immunofluorescence images of the morphological changes in mitosomes of *E*. *histolytica* by expression of GTPase-deficient EhDRP-HA. Trophozoites of each transformant strain were cultured with or without 5 µg/ml tetracyclin [Tet (+) or Tet (−), respectively] for four days. HA-tagged EhDRP mutants (red) were stained with anti-HA antibody and Alexa Fluor^®^ 594 goat anti-mouse IgG. Mitosomes (green) were stained by anti-APSK antibody and Alexa Fluor^®^ 488 goat anti-rabbit IgG. Merged images of DIC and fluorescence are also shown. These images are approximately 1.0 µm in thickness per section. Scale bar = 5 µm. Experiments were performed at least twice and more than 40 cells were observed for each condition.

It was previously shown that the expression of a GTPase-deficient Drp1 caused morphological changes in the mitochondria of human cells, as well as that of protozoan organisms^16,28^. To determine whether a similar phenomenon also occurs in *E*. *histolytica*, we performed IFA and examined the morphology of mitosomes in transformant lines which expressed GTPase-deficient EhDRP-HA upon Tet induction (Fig. 3). In the absence of Tet, the morphology of mitosomes was normal, appearing as punctate signals for the localization of APSK, in all strains [EhDrpA(K38A)-HA, EhDrpB(K39A)-HA, EhDrpC(K164A)-HA, EhDrpD(K121A)-HA, and control strains]. However, upon induction of protein expression, we observed striking elongation of mitosomes in EhDrpA(K38A)-HA and EhDrpB(K39A)-HA strains. The diameter of a typical spherical mitosome in *E*. *histolytica* trophozoites ranges from 150 to 400 nm^24^. Although the length of elongated mitosomes could not be measured precisely (because a single elongated mitosome often crosses multiple confocal planes), it often exceeded 5 µm and sometimes reached 30 µm (relative to the scale bar shown). These results can be interpreted as prevention of fission caused by the expression of either GTPase-deficient EhDrpA or EhDrpB, suggesting that both DRPs participate in the fission process of *Entamoeba* mitosomes. In this study, we also identified EhDRPs showing nuclear localization (EhDrpC and EhDrpD). Subsequent analyses were not performed on these DRPs as such were beyond the scope of this study. However, we should mention that in mice, a nucleus-localized DRP called Mx1, an interferon-induced intracellular restriction factor with known antiviral activity, has been reported^33,34^.

### Phenotypes of repression of *EhDrpA* and *EhDrpB* gene expression

**Figure 4:**
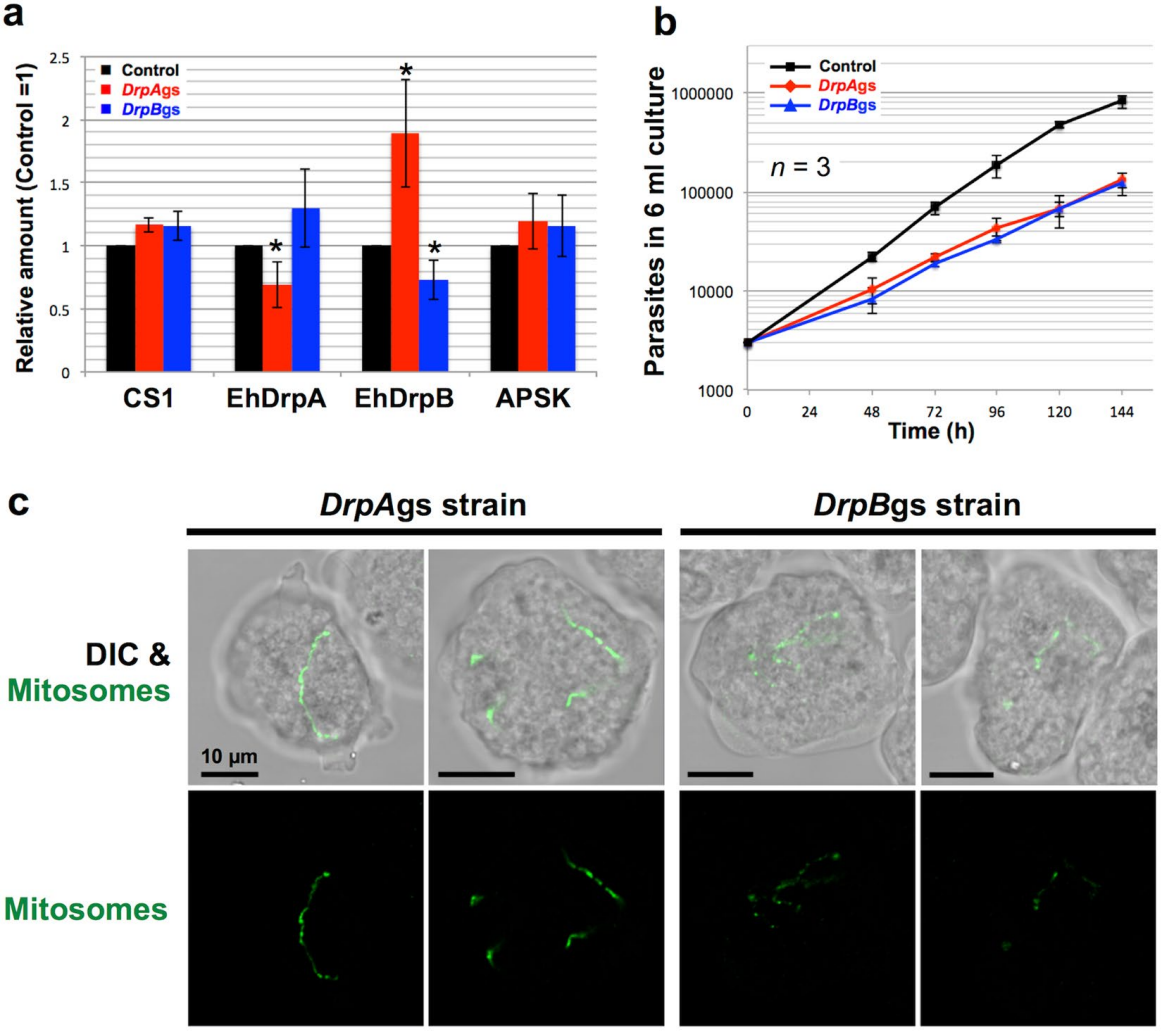
Effects of repression of gene expression of *EhDrpA* and *EhDrpB* genes. (**a**) Relative amounts of EhDrpA, EhDrpB, APSK (mitosomal matrix protein), and cysteine synthase 1 (CS1, BAA21916, as an irrelevant control gene) in the strains where *EhDrpA* or *EhDrpB* gene was silenced [*DrpA*gs (red bars) and *DrpB*gs (blue bars)]. Control (black bars) indicates the strain transfected by the empty vector. The intensities of the bands in the immunoblots (Supplementary Fig. S8) were measured and shown relative to the control. Approximately 20 µg protein per lane was subjected to SDS-PAGE. Error bars indicate standard deviations of three replicates. *P < 0.05 (Student t-test). (**b**) Growth kinetics of *DrpA*gs, *DrpB*gs, and control strains. Error bars indicate standard deviations of triplicates. (**c**) Immunofluorescence images of mitosomes in *DrpA*gs and *DrpB*gs strains by confocal microscopy. Mitosomes (green) were stained by anti-APSK antibody and Alexa Fluor^®^ 488 goat anti-rabbit IgG. DIC indicates the differential interference contrast image. These images are approximately 1.0 µm in thickness per section. Scale bar = 10 µm. Experiments were performed three times and more than 40 cells were observed for each strain.

We previously used small antisense RNA-mediated transcriptional gene silencing (gs)^21,24^ to demonstrate the importance of mitosomal proteins, e.g., enzymes for the sulfate activation pathway, metabolite channel, chaperones, and components of the protein import machinery, in *E*. *histolytica* survival and proliferation. Similarly, to demonstrate the biological importance of *Entamoeba* DrpA and DrpB, we established *E*. *histolytica* strains in which one of *EhDrpA* and *EhDrpB* genes was silenced (*DrpA*gs and *DrpB*gs strains, respectively). Gene silencing was verified by measuring the band intensities in immunoblots reacted with anti-DRP, CS1 (a cytosolic protein control), and APSK antibodies (Fig. 4a and Supplementary Fig. S8). The amount of EhDrpA and EhDrpB decreased by approximately 31 ± 18% (*p* < 0.05) and 27 ± 16% (*p* < 0.05) in *DrpA*gs and *DrpB*gs strains, respectively. Interestingly, the amount of EhDrpA seemed to increase by 30 ± 31% (*p* < 0.17) in the *DrpB*gs strain, while EhDrpB increased by 89 ± 43% (*p* < 0.05) in the *DrpA*gs strain, suggesting possible compensatory mechanisms. We also monitored the growth of *DrpA*gs and *DrpB*gs strains and observed that their growth was inhibited when compared to that of the control strain (Fig. 4b). The doubling times of the control, *DrpA*gs, and *DrpB*gs strains were 20.1 ± 0.1, 28.0 ± 1.7 (*p* < 0.01: Student t-test), and 27.0 ± 2.8 (*p* < 0.02) hours, respectively. These results suggest that both EhDrpA and EhDrpB are indispensable for normal proliferation of *E*. *histolytica* trophozoites. In addition, IFA data showed that silencing of *EhDrpA* and *EhDrpB* genes caused mitosomal elongation comparable to that caused by the expression of corresponding GTPase-deficient *EhDRP* genes (Fig. 4c).

### Potential crosstalk and physical interaction between EhDrpA and EhDrpB

**Figure 5:**
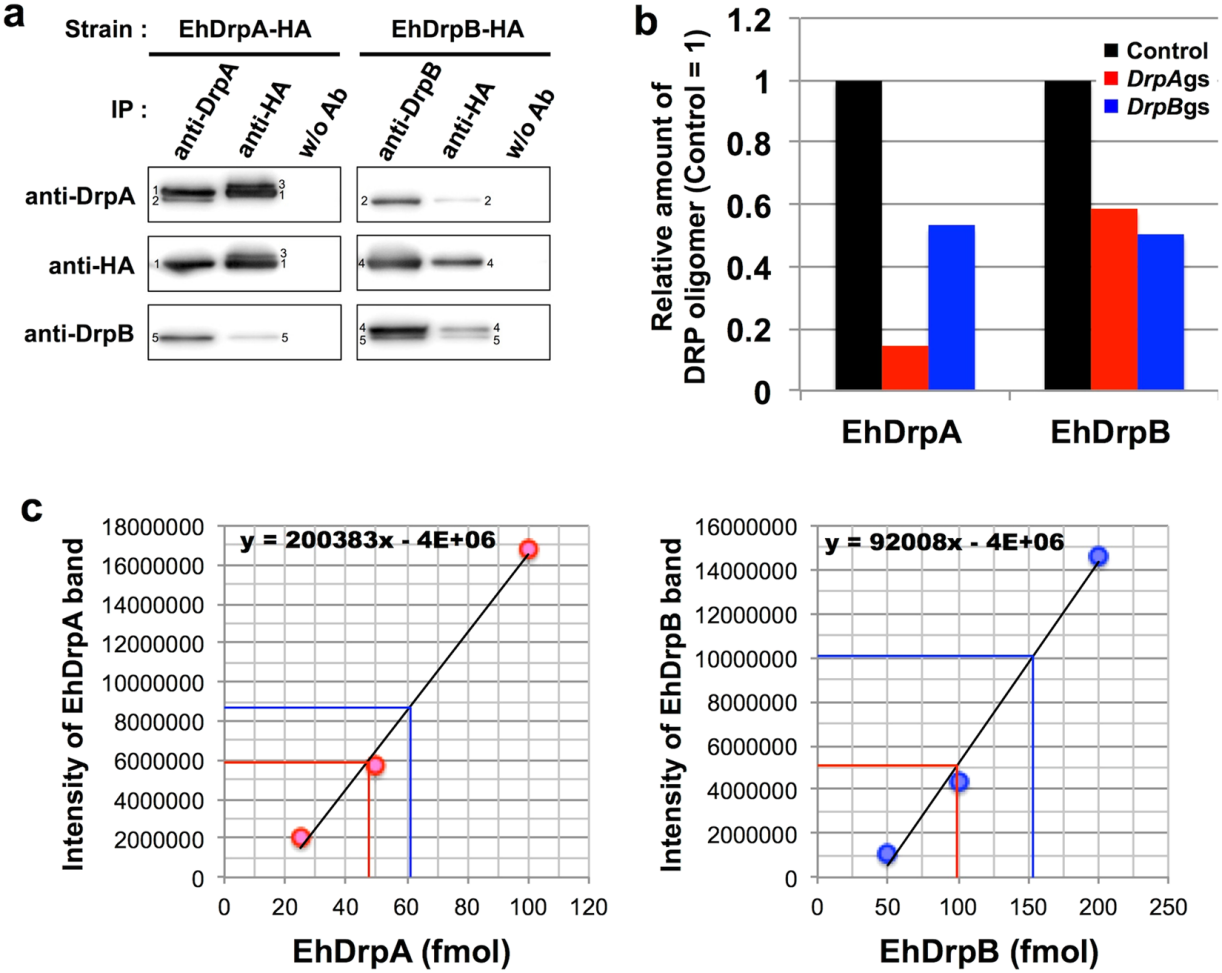
Analyses of EhDrpA-EhDrpB interaction. (**a**) Immunoprecipitation and immunoblot analysis of EhDRPs. EhDrpA and EhDrpB were immunoprecipitated from whole cell lysates (1 mg protein) of EhDrpA-HA and EhDrpB-HA strains using anti-DrpA, anti-DrpB, and anti-HA antibodies. Immunoprecipitation was also carried out without antibody (“w/o Ab”). Immunoprecipitated proteins were subjected to SDS-PAGE (about 150 µg protein per lane). Antibodies used for immunoprecipitation (IP) and immunoblots are indicated. Individual bands are labeled as follows: 1, EhDrpA-HA; 2, endogenous EhDrpA; 3, post-translationally modified EhDrpA-HA; 4, EhDrpB-HA; 5, endogenous EhDrpB. Original and unclipped immunoblots are shown in Supplementary Fig. S10. (**b**) Relative amounts of EhDrpA and EhDrpB in the 900-kDa EhDRP complex of *DrpA*gs, *DrpB*gs, and control strains. *DrpA*gs (red bars), *DrpB*gs (blue bars), and control (black bars) denote the strains in which *EhDrpA* or *EhDrpB* gene was silenced, and control strain, respectively. The intensities of the bands in the immunoblots (Supplementary Fig. S11) were measured and shown relative to the control. (**c**) A standard curve for the quantification of EhDrpA and EhDrpB in the hetero-oligomer. EhDRP was immunoprecipitated from trophozoites of *E*. *histolytica* HM-1:IMSS cl6 wild type by anti-DrpA or anti-DrpB antibodies. Shown on the horizontal axis are the amounts of each EhDRP (in fmol). On the vertical axis, the intensity of EhDrpA and EhDrpB bands in the immunoblots are shown respectively (see also Supplementary Fig. S12). Circles indicate the recombinant histidine-tagged (His_6_)-EhDRP-HA proteins of various known amounts used as standards, and measured for band intensities in immunoblots using specific antibodies against EhDRPs. Standard curves were created by linear regression. Red and blue lines indicate the intensities of the detected EhDrpA and EhDrpB bands in immunoblots of the immunoprecipitated samples by anti-DrpA and anti-DrpB antibodies, and the estimated amount of EhDrpA and EhDrpB, respectively.

EhDrpA and EhDrpB have significant sequence identity at the amino acid level (44%, Supplementary Fig. S9) and distribute to similar cellular localization (both the cytoplasm and mitosomes, Fig. 2a). Expression of a mutant form or gene silencing of these EhDRPs elicited similar morphological and growth defects (Figs 3, 4b,c, and Supplementary Fig. S7). Although these facts may indicate that they may perform overlapping functions, it is not the case. EhDrpA cannot complement a defect caused by gene silencing of EhDrpB, and vice versa (Fig. 4). These observations led us to the premise that EhDrpA and EhDrpB may have physical and/or genetic interactions and be coordinately involved in mitosome fission. Thus, we attempted to verify whether a complex is formed via physical interaction between EhDrpA and EhDrpB by immunoprecipitation of native and HA-tagged EhDRPs from EhDrpA-HA and EhDrpB-HA strains, followed by immunoblot analysis (Fig. 5a). EhDrpA was co-immunoprecipitated by anti-HA and anti-DrpB antibodies from whole cell lysates of EhDrpB-HA strain. Conversely, EhDrpB was co-immunoprecipitated by anti-HA and anti-DrpA antibodies from whole cell lysates of EhDrpA-HA strain. These data suggest that there is physical interaction between EhDrpA and EhDrpB in *E*. *histolytica* trophozoites. To confirm the importance of this interaction, we performed blue native polyacrylamide gel electrophoresis using whole cell lysates of *DrpA*gs, *DrpB*gs, and control strains followed by immunoblot analysis using anti-DrpA and anti-DrpB antibodies (Supplementary Fig. S11). An approximately 900-kDa protein band was detected in all samples. The intensities of the band were compared among the three strains (Fig. 5b), showing that gene silencing of EhDrpA caused a decrease in the incorporation of EhDrpB into a 900-kDa complex, and conversely, gene silencing of EhDrpB decreased the EhDrpA incorporation into the complex. These data strongly suggest that both EhDrpA and EhDrpB are indispensable for the formation and the maintenance of the DRP oligomer. We further calculated the molecular ratio of EhDrpA and EhDrpB in the oligomer. The samples immunoprecipitated by anti-DrpA or anti-DrpB antibodies were subjected to immunoblot analysis, using histidine-tagged (His_6_)-EhDrpA-HA and His_6_-EhDrpB-HA recombinant proteins (Supplementary Fig. S12a) as standards (Supplementary Fig. S12b). Immunoprecipitation of the EhDrpA/B complex with anti-DrpA antibody yielded 48.9 fmol of EhDrpA and 98.8 fmol of EhDrpB (Fig. 5c). Similarly, immunoprecipitation of the complex with anti-DrpB antibody resulted in the coprecipitation of 61.5 fmol of EhDrpA and 153.6 fmol of EhDrpB (Fig. 5c), indicating that the molecular ratio of EhDrpA and EhDrpB is likely 1:2 to 1:3.

## Discussion

We have demonstrated that two DRPs, EhDrpA and EhDrpB, are cooperatively involved in the mitosomal fission in *E*. *histolytica* (Figs 2–5). A line of evidence suggests that the fission of *Entamoeba* mitosomes is mediated by the hetero-oligomer of these two DRPs. To our knowledge, this is the first report of two DRPs coordinately engaged in mitochondrial fission. Moreover, our phylogenetic analysis showed that DrpAs and DrpBs from various *Entamoeba* species form independent clades with high bootstrap support (Fig. 1), suggesting that gene duplication resulted in DrpA and DrpB in an *Entamoeba* common ancestor prior to *Entamoeba* speciation (at least before separation of *E*. *histolytica/E*. *dispar* and *E*. *invadens*). However, it remains to be demonstrated whether DrpA-DrpB hetero-oligomer formation occurs in all *Entamoeba* lineages.

Mitofusin (Mfn) and fuzzy onion are DRPs known to be involved in the fusion of the mitochondrial outer membrane in metazoan and fungal cells, respectively^35^. Mouse Mfn1 and Mfn2 were reported to form both homo- and hetero-oligomers^36,37^. The interaction between EhDrpA and EhDrpB suggests that three kinds of mechanochemical rings are potentially formed by these two DRPs: two homo- and one hetero-oligomer structures. In the previous *in vitro* study, it was shown that EhDrpA (therein named as EhDlp1^26^) forms a homo-oligomer and has activity to change the morphology of spherical liposomes into tubular form^26^. However, the molecular ratio of EhDrpA and EhDrpB estimated by co-immunoprecipitation and immunoblot analysis (Fig. 5) strongly suggests that these EhDRPs mainly exist as hetero-oligomers *in vivo*.

One puzzling question is why *E*. *histolytica* (and other *Entamoeba* species) use two distinct DRPs for the fission of mitosomes. One possible reason is that two DRPs make the constriction helical ring of DRP small enough to fit the size of *Entamoeba* mitosomes. Yeast Dnm1 was predicted to form a homo-oligomeric helical ring of which the outer diameter on liposomes is 129 nm^38^. Moreover, the diameter of mitochondrial constriction sites observed was reported as 109 ± 24 nm^39^. Although there is no published data available on the size of the constriction site in mitosomes, the size of mitosomes *per se* is smaller than that of canonical mitochondria (the diameter of *E*. *histolytica* mitosomes is 150–400 nm^24^). Our preliminary size estimation using electron micrographs (Supplementary Fig. S13) indicates that the diameter of constriction-like structures is 77.6 ± 19.2 nm (n = 11). Therefore, the hetero-oligomeric spiral composed of EhDrpA and EhDrpB with 1 to 2–3 ratio, which likely forms the constriction ring with the size and structure different from the model organism, is probably required for coiling around “smaller” mitosomes of *Entamoeba*. The second possible reason is that the hetero-oligomeric complex may be involved in the regulation of mitosomal fission during cell cycle or stage conversion of *Entamoeba* species. This possibility seems to be supported by the transcriptome data of *Entamoeba invadens* ^40,41^, a model species for *Entamoeba* stage conversion. *E*. *invadens* possesses a single gene for EhDrpA homolog (EIN_051430) and two genes for EhDrpB homolog (EIN_070060 and EIN_254810). Early in excystation, these *E*. *invadens DRP* genes were 10–30 fold up-regulated^40^ (Supplementary Fig. S14). In addition, genes encoding two isotypes of EhDrpB homologs were down-regulated in the early (~24 hours) phase of encystation^41^. The imbalance in EhDrpA and EhDrpB homologs likely cause an interruption of mitosome fission during encystation. These data on the coordinated expression regulation of *DrpA* and *DrpB* genes are also consistent with the close physical and genetic interaction between the two proteins.

It was demonstrated with mammalian Drp1 that its GTPase activity, oligomer formation, affinity to DRP receptors, and stability on the mitochondrial membrane, were regulated by post-translational modifications^7^. Similarly, it is plausible that EhDrpA and EhDrpB are transcriptionally and post-translationally regulated and such regulations play an important role in mitosome fission during proliferation and stage conversion. EhDrpA and EhDrpB indeed possess putative modification sites for phosphorylation and sumoylation, as predicted by NetPhos and SUMOplot programs, respectively (Supplementary Fig. S9). Moreover, putative post-translational modification sites also exist in the region selected to raise anti-DrpA antibody (Supplementary Fig. S9). The result of our immunoprecipitation with anti-DrpA and anti-HA antibodies showed an additional EhDrpA-HA band (possibly post-translationally modified), which was undetectable by anti-DrpA antibody in the lane of EhDrpA-HA strain (Fig. 5a), suggesting that the epitope may be hidden from anti-DrpA antibody by post-translational modifications. Phosphorylation of EhDrpA and EhDrpB was suggested by immunoblot analysis using acrylamide gel containing Phos-tag which has an affinity to the phosphate base on the surface of proteins (Supplementary Fig. S15). Our preliminary immunoprecipitation experiment did not suggest any interaction between the small ubiquitin-related modifier (SUMO; XP_655984)^42^, which was expressed as an HA epitope-tagged protein at the amino terminus, and EhDrpA or EhDrpB using anti-HA, anti-DrpA, and anti-DrpB antibodies (data not shown). However, we cannot exclude the possibility that SUMOs bound to EhDrpA or EhDrpB were cleaved from EhDRPs by SUMO-specific protease activity since the protease activity could not be completely inhibited during immunoprecipitation and/or that the amount of sumoylated EhDRPs is below the detection limit by immunoblotting. Further study is necessary to verify post-translational modifications of EhDrpA and EhDrpB and whether such modifications are required for the regulation of mitosomal fission in *Entamoeba* species.

We have shown that mitosomes are dramatically elongated by expression of a dominant negative GTPase-deficient form of EhDrpA or EhDrpB or the knockdown of wild type *EhDRP* genes (Figs 3 and 4c). It is important to note that the elongation of mitosomes was accompanied with growth defect (Fig. 4b), suggesting that proper regulation of mitosomal fission is essential for cell proliferation. However, we cannot exclude the possibility that this growth retardation was caused by a defect in other functions besides mitosomal fission in which EhDrpA and EhDrpB are involved. Jain *et al*. previously reported that EhDrpA accumulated at the periphery of the nucleus and proposed that EhDrpA may contribute to the division of the nucleus in *Entamoeba*, where the nuclear membrane did not disintegrate during nuclear division^26^, although we were unable to reproduce their findings in the present study. In addition, it has also been reported in other uni- and multicelullar eukaryotes that mitochondrial DRPs participate in the cleavage of other organellar membrane^28,35^.

Phylogenetic analysis indicates that mitosome-associated DRPs in *Entamoeba* are largely divergent from the *Trichomonas* Drp (XP_001305587)^16^, which is involved in the fission of hydrogenosomes (Fig. 1). Although DRPs in *Entamoeba* and *Trichomonas* seem to be only distantly related, which likely reflects taxonomic separation between Amoebozoa and Excavata supergroups, the fission of their divergent MROs requires analogous DRPs, similar to the case of canonical aerobic mitochondria. Furthermore, *Entamoeba* also seems to have invented a unique hetero-oligomerization of two DRPs in a lineage-specific manner. To finally validate our observation on the involvement of EhDrpA and EhDrpB in mitosome fission, a close examination of division and biogenesis of mitosomes throughout the cell cycle is needed, although the checkpoints of cell cycle in *E*. *histolytica* are promiscuous and have not yet been demonstrated^43,44^. It is also worth mentioning that there is a possibility that the elongation phenotype may be caused indirectly by mutant expression or gene silencing of EhDRPs via yet-uncharacterized mechanisms including protein transport to mitosomes, cytoskeletal rearrangement, and cytokinesis. Our finding shall provide a new example of structural diversity of DRP oligomers and helps in the elucidation of the mechanical diversity of mitochondrial dynamics.

## Methods

### Organisms and culture

Trophozoites of *Entamoeba histolytica* HM-1:IMSS cl6^45^ and G3^46^ strains were cultivated axenically in Diamond BI-S-33 medium as previously described^47^.

### RNA and cDNA preparation

Isolation of total RNA and mRNA, and synthesis of cDNA were performed as previously described^21^.

### Plasmid construction

To generate the Tet-inducible expression vector with the neomycin resistance marker (pEhTex/HA, see Supplementary Fig. S4), the region of TetR - TetO was amplified from pEhHygtetR-O-Cass vector^48,49^, a kind gift from Dr. William A. Petri, Jr., using Phusion DNA polymerase (New England Biolabs) and corresponding primers (Supplementary Table S2). After purification, the amplified fragment was ligated into pEhEx/HA^50^ digested by *Bgl* II using In-Fusion^®^ HD Cloning Kit (TaKaRa Bio Inc., Shiga, Japan).

For Tet-dependent expression of HA-tagged proteins, the *E*. *histolytica* genes used in this study were PCR-amplified from cDNA using Phusion DNA polymerase and corresponding primer sets (Supplementary Table S2). After restriction digestion and purification, the amplified fragments were ligated into pEhEx/HA or pEhTex/HA digested by *Bgl* II using Ligation-Convenience Kit (Nippon gene Co., Tokyo, Japan) or In-Fusion^®^ HD Cloning Kit.

For gene silencing, 357- and 327-bp fragments corresponding to the amino terminus of EhDrpA and EhDrpB, respectively, were PCR-amplified with appropriate primers (Supplementary Table S2). The fragments were digested using *Stu* I and *Sac* I, and ligated into *Stu* I/*Sac* I double-digested psAP-2-Gunma plasmid^24^.

### Amoeba transformation

Lipofection of trophozoites, selection, and maintenance of transformants were performed as previously described^19^.

### Peptide antibodies

We prepared *in vitro* synthetic peptides “CIPQQPTTKPPKKQSPSK (521–537 amino acid position in EhDrpA)” and “QAKPQQQHVPKESITTSC (542–558 amino acid position in EhDrpB)”, as antigens to raise antisera against EhDrpA and EhDrpB, respectively (see also Supplementary Fig. S9). After the conjugation of keyhole limpet hemocyanin (SIGMA) by m-maleimidobenzoyl-N-hydroxysuccinimide ester (PIERCE), antigens were immunized into independent rabbits (Japanese white rabbit). After booster injections with the same antigens, whole blood was collected from immunized rabbits and antisera were separated from blood. Anti-EhDRP antibodies were purified by affinity columns conjugated with the synthetic peptide.

### Recombinant proteins

To generate recombinant His_6_-EhDrpA-HA and His_6_-EhDrpB-HA proteins, we amplified genes using appropriate primers sets (Supplementary Table S2) and pEhEx/EhDrpA-HA and pEhEx/EhDrpB-HA as templates. Fragments were digested by appropriate sets of restriction enzymes and ligated into pCold™ I plasmid (TaKaRa). These plasmids were transformed into BL21 Star™ (DE3) One Shot^®^ Chemically Competent *E*. *coli* (Invitrogen) and expression of recombinant proteins was induced by low temperature (15 °C) with 1 mM IPTG. After induction, target proteins were purified from bacterial lysates by Ni-NTA system (QIAGEN GmbH, Hilden, Germany).

### Immunoblot assay

After running SDS-PAGE using 5–20% gradient polyacrylamide gel (ATTO CORPORATION, Japan), the proteins in gel were transferred onto Amersham^TM^ Hybond^TM^ P 0.45 PVDF membrane (GE Healthcare). The membranes were incubated with 5% skim milk in PBST for blocking.

For blue native polyacrylamide gel electrophoresis (BN-PAGE), *DrpA*gs, *DrpB*gs, and their control strains were solubilized by 1% digitonin (Invitrogen) at 4 °C for 30 min, and centrifuged at 20,000 × *g* for 30 min at 4 °C. BN-PAGE was performed using NativePAGE^TM^ Novex^®^ Bis-Tris Gel System (Invitrogen) according to manufacturer’s protocol. The digitonin concentration in samples was adjusted to 0.5% before electrophoresis.

Anti-HA mouse monoclonal antibody (HA.11 16B12, COVANCE) was diluted 1000 fold with PBST containing 5% skim milk. Anti-CS1^51^, anti-DrpA, anti-DrpB, and anti-APSK^19^ rabbit antibodies were prepared as described previously, and diluted 500-, 500-, 200-, and 1000-fold with 5% skim milk in PBST, respectively. Anti-mouse and anti-rabbit immunoglobulin F(ab’)_2_ fragment conjugated with horseradish peroxidase (Amersham) were diluted 3000 times with PBST and used as secondary antibody. Immobilon^TM^ Western (MILLIPORE) was used as a substrate for visualization of the proteins. Detection of chemiluminescence and quantification of band intensities were performed by Ez-Capture MG and CS Analyzer ver 3.0 (Atto Co., Tokyo, Japan), respectively.

### Immunofluorescence assay

Sample preparation for immunofluorescence assay was performed as previously described^19,52^. Briefly, we used PEM buffer (100 mM PIPES-NaOH/2 mM EGTA/1 mM MgSO_4_, pH 6.9) for all steps. PEM buffer containing 25% (v/v) acetone/25% (v/v) methanol was used to fix cells for observing the localization of EhDRPs. We also used PEM buffer containing 3.7% paraformaldehyde to examine morphological changes in mitosomes. Confocal fluorescence images were captured and analyzed by the LSM510 Meta confocal Microscope (Carl Zeiss) with lambda (emission fingerprinting mode^53,54^) and the ZEN 2009 software.

### Immunoprecipitation

Whole cell lysates were solubilized with 50 mM Tris-HCl/150 mM NaCl /1% Triton X-100 (pH 7.4) with protease inhibitors. The lysate was mixed with Protein G Magnetic Beads (New England Biolabs) and anti-HA, anti-DrpA, or anti-DrpB antibodies overnight at 4 °C. The beads were washed four times with 50 mM Tris-HCl/150 mM NaCl/1% Triton X-100 (pH 7.4) at 4 °C. Bound proteins were released from the beads by mixing with SDS sample loading buffer containing 5% 2-mercaptoethanol and boiling at 95 °C for 3 min.

### *In silico* analyses

To survey *Entamoeba* candidate proteins involved in the fission and fusion of mitosomes in AmoebaDB, we performed BLASTP search using sequences of full-length proteins known to be involved in mitochondrial fission in human, yeast, and *Dictyostelium*, as query. Survey was also conducted using consensus sequences of domains (e.g. dynamin-GTPase domain) generated by the hidden Markov model in the Pfam database (URL: http://pfam.xfam.org), as query. After acquiring amino acid sequences of candidates from AmoebaDB, the sequences were used as a query for Pfam and BLAST search to identify human/yeast/*Dictyostelium* homologs (https://www.ncbi.nlm.nih.gov/mapview/). Finally, we excluded candidates if they were found by Pfam (cut-off: *E*-value < 10.0) to lack the domain present in the human/yeast/*Dictyostelium* conterparts and/or if no human/yeast/*Dictyostelium* homolog was identified by BLASTP search.

Prediction for the presence of transmembrane regions was performed using TMHMM (http://www.cbs.dtu.dk/services/TMHMM-2.0/), HMMTOP (http://www.enzim.hu/hmmtop/index.php), and SOSUI (http://harrier.nagahama-i-bio.ac.jp/sosui/sosui_submit.html) programs. cNLS Mapper (http://nls-mapper.iab.keio.ac.jp/cgi-bin/NLS_Mapper_form.cgi) was used to predict the importin α-dependent nuclear localization signal. Putative phosphorylation and sumoylation sites in EhDrpA and EhDrpB were predicted by NetPhos (http://www.cbs.dtu.dk/services/NetPhos/) and SUMOplot (http://www.abgent.com/sumoplot) programs, respectively.

### Phylogenetic analysis

Multiple alignment of the sequences of dynamin superfamily proteins was obtained using MUSCLE (http://www.ebi.ac.uk/Tools/msa/muscle/)^55^, and was corrected by manual inspection. Unambiguously aligned positions were selected and used for phylogenetic analyses, which was performed by the maximum likelihood method using the LG model^56^ with four categories of among-site rate variation and the rate variation model allowed for some sites to be evolutionarily invariable implemented in the MEGA7 software^57^. With 83 proteins from 20 species, 350 unambiguously aligned amino acid sites were used for the analysis, corresponding to residues 3–13, 24–54, 58–72, 91–100, 119–138, 156–164, 171–174, 182–193, 196–211, 214–220, 229–235, 237–239, 247–250, 255–257, 260–264, 270–304, 309–314, 317–339, 364–370, 387–390, 399–405, 410–414, 420–430, 438–452, 455–468, 472–475, 587–603, 605–620, 623–628, 644–663, and 667–669 of the EhDrpA sequence. All alignments are available from the authors upon request.

## Electronic supplementary material

Supplementary information
