# Supplementary material for: Hetero-oligomer of dynamin-related proteins participates in the fission of highly divergent mitochondria from Entamoeba histolytica
Source: Sci Rep. 2017 Oct 18;7:13439. doi: 10.1038/s41598-017-13721-5 (PMC5647421; doi:10.1038/s41598-017-13721-5)
Supplement: Supplementary file 1 — Supplementary information [file 41598_2017_13721_MOESM1_ESM.pdf]

Supplementary information:

Hetero-oligomer of dynamin-related proteins participates in the fission of highly divergent mitochondria from *Entamoeba histolytica*

Takashi Makiuchi [a*], Herbert J. Santos [b], Hiroshi Tachibana [a], and Tomoyoshi Nozaki [b,c,d*]

[a] Department of Infectious Diseases, Tokai University School of Medicine, 143 Shimokasuya, Isehara, Kanagawa 259-1193, Japan; [b] Department of Parasitology, National Institute of Infectious Diseases, 1-23-1 Toyama, Shinjuku-ku, Tokyo 162-8640, Japan; [c] Graduate School of Life and Environmental Sciences, University of Tsukuba, 1-1-1 Tennodai, Tsukuba, Ibaraki 305-8572, Japan; [d] Department of Biomedical Chemistry, Graduate School of Medicine, the University of Tokyo, 7-3-1 Hongo, Bunkyo-ku, Tokyo 113-0033, Japan

* Corresponding authors: T. Makiuchi and T. Nozaki

TM; E-mail: makky@tokai-u.jp; Phone: +81(Japan)-463-93-1121 ext. 2600; Fax: +81-463-95-5450

TN; E-mail: nozaki@m.u-tokyo.ac.jp; Phone: +81(Japan)-3-5841-3526; Fax: +81-3-5841-3444

Legends of Supplementary Figures

Supplementary Fig. S1

Domain prediction of EhDRPs by Pfam. The human (*Homo sapiens,* Hs) Drp1 (NP_036192), which is involved in mitochondrial fission, was predicted to contain a dynamin family GTPase domain (green box), a middle domain (red box), and a GTPase effector domain (blue box) based on Pfam analysis. The same three domains were likewise detected in human dynamin 1 (HsDyn1: NP_004399) in addition to the pleckstrin homology (PH) domain (orange box), whereas only the dynamin family GTPase domain was predicted in human Opa1 (HsOpa1: AAH75805). HsOpa1, a protein involved in mitochondrial inner membrane fusion, also contains a mitochondrial targeting sequence (MTS) and a transmembrane region (TM). *E. histolytica* possesses four genes for DRPs (EhDrpA, EhDrpB, EhDrpC, and EhDrpD) in its genome. The previously reported *E. histolytica* DLP1 (dynamin-like protein 1)[1], is designated here as EhDrpA for uniformity among the four EhDRPs in this study. The corresponding *e*-values calculated by the Pfam prediction are indicated in the boxes. The amino acid stretch depicted in EhDrpC corresponds to the putative nuclear localization signal predicted by the cNLS Mapper.

Supplementary Fig. S2

Confirmation of the expression of HA-tagged EhDrpA-D by immunoblot assay. The left panel shows the immunoblot reacted with anti-HA mouse monoclonal antibody (HA.11 16B12), while the right panel corresponds to the blot stained with anti-CS1 rabbit antibody (as a loading control)[2]. Predicted sizes of EhDrpA-HA, EhDrpB-HA, EhDrpC-HA, and EhDrpD-HA are about 80.5-, 78.3-, 94.6-, and 94.1-kDa, respectively. Each lane contains 10 µg protein.

Supplementary Fig. S3

Confocal immunofluorescence images and fluorescence intensity profiles of *E. histolytica* transformants expressing EhDRP-HA. These images were acquired as shown in Fig. 2. The line color in graphs on the bottom panels corresponds to the respective fluorescence color in immunofluorescence images. Scale bar = 10 μm. Bottom graphs show the profile of fluorescence intensities on the line from the merged fluorescence micrographs. Each image of EhDrpC-HA and EhDrpD-HA strains on the left is identical to one shown in Fig. 2.

Supplementary Fig. S4

Scatter plots of the distribution of fluorescence signals for the evaluation of co-localization between EhDRP-HA and APSK or TOTO-3. The plots were created and analyzed using the "Coloc" option in Imaris® software (Ver. 6. 2. 1, Bitplane). The Diagrams shown in red and blue boxes were generated from images in Fig. 2 and Supplementary Fig. S3, respectively. The diagrams in yellow and green boxes were generated from images of EhDrpC-HA and EhDrpD-HA strains shown on the right side in Supplementary Fig. S3, respectively. Horizontal and vertical axes for each diagram are depicted at the bottom and top of the figure. Values (r) in parentheses indicate Pearson correlation coefficient.

Supplementary Fig. S5

(a) Comparison of DRPs between human and *E. histolytica*. Multiple alignment of a partial GTPase domain in EhDRPs, together with the human Drp1. The lysine residue (K) that plays an important role in the GTPase activity is highlighted[1,3]. This residue was mutated into alanine to create GTPase-deficient DRPs. Asterisks and dots indicate identical and similar amino acid residues, respectively. (b) Verification of the expression of GTPase-deficient mutants of EhDrpA,

B, C, and D. Expression of HA-tagged mutant EhDRPs was induced with 5 μg/ml Tet. Whole cell lysates (15 μg protein) of each strain cultured with (+) or without (-) Tet were subjected to SDS-PAGE and immunoblot analysis with either anti-HA antibody (left panel) or anti-CS1 antibody (right panel). The expression of EhDrpD(K121A)-HA was leaky as it was observed even in the absence of Tet induction (see also Supplementary Fig. S5c). (c) Multiple alignment of the amino terminus of DrpD among five *Entamoeba* species. Two potential initiation codons, as highlighted in green, are conserved in *Entamoeba* DrpDs. Asterisks and dots indicate identical and similar amino acid residues, respectively.

Supplementary Fig. S6

Construction scheme and map of the tetracycline (Tet)-inducible expression vector with neomycin resistance marker: pEhTex/HA. The upper panel shows a schematic diagram summarizing the construction process of pEhTex/HA. The black boxes labeled Neo and Hyg represent resistance genes for neomycin and hygromycin, while the pink boxes labeled TetR and TetO indicate the gene of the Tet repressor and operator sequences, respectively. The gray boxes labeled Lec5' and 3', the white boxes labeled Actin 5' and 3', and the yellow boxes labeled CS 5' and 3', indicate the positions of the 5'- and 3'-untranslated regions (UTR) of Lec (a heavy subunit of Gal/GalNAc lectin: XP_656181), actin, and CS1 genes, respectively. The region containing TetR to TetO of pEhHygtetR O cass vector[4,5] was PCR amplified using specific primers (pink arrows) containing the sequence needed for the In-Fusion system (broken lined boxes) at each 5' end. The red boxes represent the sequence of three copies of the hemagglutinin (HA) tag. The amplified fragment was inserted into pEhEx/HA[6] digested with *Bgl* II. The lengths in the scheme are not proportional to the actual numbers of nucleotides. The lower panel indicates the nucleotide sequence near the cloning site of pEhTex/HA. The newly constructed vector

pEhTex/HA has three main features absent in pEhHygtetRO cass vector. First, the cloning sites in pEhEx[5] and pEhTex/HA are interchangeable. Second, selection after transfection is by neomycin treatment, a more convenient approach compared with hygromycin selection. Third, like pEhEx[7], pEhTex/HA can be used for the expression of a second protein by cloning in the single *Spe* I site.

Supplementary Fig. S7

Growth kinetics of *E. histolytica* trophozoites expressing a GTPase-deficient DRP mutant. Transformants were cultured with [Tet (+)] (red) or without [Tet (-)] (blue) 5 μg/ml Tet, and cell densities were monitored at indicated time points. Error bars represent standard deviations of three replicates. * $P < 0.05$ (Student's t-test).

Supplementary Fig. S8

(a) Detection of EhDrpA and EhDrpB in whole cell lysate and immunoprecipitated samples by anti-EhDRP antibodies. Lane 1: immunoprecipitated (IP) sample by antiserum against DrpB. Lane 2: IP sample by antiserum against DrpA. Lane 3: IP sample by anti-DrpB antibody. Lane 4: IP sample by anti-DrpA antibody. Lane 5: IP sample by anti-HA antibody. Lane 6: mock IP sample carried out without antibody. Lane 7: whole trophozoite lysate (WTL) of *E. histolytica* HM-1:IMSS cl6. Approximately 20 μg of WTL protein or approximately 150 μg of immunoprecipitated protein per lane were subjected to SDS-PAGE and immunoblot analysis (left and right panels are results of anti-DrpA and anti-DrpB antibodies, respectively). Anti-DrpA and anti-DrpB antibodies can detect EhDrpA and EhDrpB as a major band in the lanes loaded with IP sample by anti-DrpA and anti-DrpB antibodies, respectively, although anti-DrpB antibody has nonspecific reaction in WTL lane. Moreover, the reactivity of anti-DrpB antibody to EhDrpB was

also demonstrated in the immunoblots of strains expressing EhDrpB-HA (Supplementary Fig. S10) as well as in recombinant EhDrpB (Supplementary Fig. S12b). (b) Comparison of protein expression among control, *DrpA*gs, and *DrpB*gs strains. Fresh samples for immunoblot analysis were prepared from three independent cultures. DrpA, DrpB, APSK, and CS1 indicate immunoblots reacted with anti-DrpA, anti-DrpB, anti-APSK, and anti-CS1 antibodies, respectively. Membranes in the 1st trial were cut prior to reaction with respective antibodies. The 2nd and 3rd trials were performed at the same time, following a similar procedure to that of the 1st trial, with the samples loaded on the same gel. Approximately 20 μg of WTL protein per lane was subjected to SDS-PAGE. Bands marked with arrowheads indicate target proteins and band intensities for Fig. 4a were measured using the bands in Supplementary Fig. S8b.

Supplementary Fig. S9

Alignment of mitosome-associated EhDRPs and putative post-translational modification sites. The asterisks indicate conserved amino acids. Prediction of phosphorylation sites was performed using the NetPhos program (http://www.cbs.dtu.dk/services/NetPhos/). Residues in red, blue, and green are the predicted phosphorylation sites with high (score > 0.9), intermediate (0.8-0.9), and low (< 0.8) probabilities respectively. Putative sumoylation motifs with high (red boxes) and low (blue boxes) probabilities were predicted using the SUMOplot program (http://www.abgent.com/sumoplot). The regions used to raise anti-DRP antibodies are highlighted in gray.

Supplementary Fig. S10

Immunoprecipitation of EhDrpA and EhDrpB from EhDrpA-HA and EhDrpB-HA strains. The *E. histolytica* line transfected with pEhEx/HA empty vector was used as control. EhDRPs were

immunoprecipitated from 1 mg proteins of whole cell lysates of each strain. W.B. denotes antibodies that were used in the immunoblot assay. IP indicates antibodies or antisera used for immunoprecipitation. Immunoprecipitation was also carried out without antibody (“w/o Ab”). Broken boxes were clipped and magnified as shown in Fig. 5a. Approximately 150 μg of immunoprecipitated protein per lane was subjected to SDS-PAGE and immunoblot analysis. Immunoblot assays using strains expressing EhDrpA-HA or EhDrpB-HA were carried out independently from those using control strain. Individual membranes were stained with either anti-HA, anti-DrpA, or anti-DrpB antibodies respectively.

Supplementary Fig. S11

Immunoblot analysis of EhDRP complex in *DRP*gs strains. Lysates of trophozoites from *DrpA*gs, *DrpB*gs , and control transformants were subjected to BN-PAGE. After blotting, the samples were reacted with either anti-DrpA or anti-DrpB antibodies. Lanes were labeled as *DrpA*gs and *DrpB*gs, for the strains in which either *EhDrpA* or *EhDrpB* gene was silenced, and control for the transformant transfected with an empty vector. The arrowhead indicates the major band (about 900-kDa) that was used as reference to compare the amount of DRP oligomer among strains. The intensities of the bands on immunoblots were measured and the relative amounts of the proteins were estimated as shown in Fig. 5b. Each lane contains 20 μg protein.

Supplementary Fig. S12

Quantification of EhDrpA and EhDrpB using recombinant histidine (His_6)-tagged EhDRP-HA proteins as standards. (a) Coomassie Brilliant Blue (R-250) staining of recombinant His_6-EhDRP-HA proteins purified from bacteria. Each lane contains 5 pmol recombinant protein. (b) Immunoblot analyses for the quantification of EhDRPs. Anti-DrpA and anti-DrpB antibodies

were used to immunoprecipitate the EhDrpA-EhDrpB complex from *E. histolytica* trophozoites. One mg protein from whole cell lysates was used in immunoprecipitation (IP). Each lane of SDS-PAGE was loaded with 3 %, 9 %, and 9 % (in volume) of the total immunoprecipitated samples with anti-DrpA or anti-DrpB antibody, and without antibody, respectively. Intensities of the corresponding EhDRP bands in immunoblots were measured and the amounts/molecular numbers of the proteins were estimated using standard curves shown in Fig. 5c.

Supplementary Fig. S13

The constriction site-like structures (CLSs) on the *E. histolytica* mitosome. (a) Immunoelectron micrograph of an *E. histolytica* mitosome with a CLS. (b) Yellow lines indicate the positions used for measuring the CLSs and the diameter of mitosomes. (c) A representative image of *E. histolytica* mitosomes. Immunoelectron microscopic observation was performed as previously described[8-10]. Gold particles indicate the localization of chaperonin 60 (Cpn60), a marker protein of *E. histolytica* mitosomes. Scale bar = 200 nm. (d) Comparison of lengths of CLSs (n = 11) and mitosome diameter (n = 22). Error bars and p-values denote standard deviations and the result of Student's t-test, respectively.

Supplementary Fig. S14

Expression level of DrpA and DrpB homologs during stage conversion of *E. invadens*. (a) Expression profile of EhDRP homologs during encystation. This profile was based on our previous paper published by De Cadiz, A. E., *et al*[11]. The horizontal axis shows the time (in hours) after induction of encystation, while the vertical axis demonstrates the fold changes of the transcripts of *E. invadens DRP* homologs relative to their expression level at the trophozoite stage (i.e., Time 0). The broken lines denote the level of EhDrpA (red) and EhDrpB (blue)

transcripts that presented the mitosome elongation phenotype in *EhDrpA* and *EhDrpB* gene-silenced strains as shown in Fig. 4. (b) The transcript levels of *E. invadens DRP* homologs relative to the trophozoite stage at 2 hours post-induction of excystation. This profile was based on a previous paper published by Ehrenkaufer, G. M. *et al*[12].

Supplementary Fig. S15

Immunoblot analysis of phosphorylated EhDrpA and EhDrpB. Strains expressing EhDrpA-HA, EhDrpB-HA, and control transformant (transfected with an empty vector), were used in this analysis. Immunoprecipitation by anti-HA antibody was performed with PhosSTOP® phosphatase inhibitor cocktail (Roche). After immunoprecipitation, the proteins were separated by SDS-PAGE using 6% acrylamide gel containing 50 µM Phos-tag™ (Wako Pure Chemical Industries, Ltd., Osaka, Japan) followed by immunoblot analysis using anti-HA antibody. Closed and open arrowheads indicate unmodified and phosphorylated EhDrpA-HA (red) and EhDrpB-HA (blue) respectively. Red and blue asterisks indicate His_6-EhDrpA-HA and His_6-EhDrpB-HA treated with AcTEV™ protease (Life Technologies) respectively, however the His-tag was not cleaved by this treatment.

Supplementary Table. S1

List of proteins known to be involved in mitochondrial dynamics[13-17]

Supplementary Table. S2

List of primers

References

1 Jain, R., Shrimal, S., Bhattacharya, S. & Bhattacharya, A. Identification and partial characterization of a dynamin-like protein, EhDLP1, from the protist parasite *Entamoeba histolytica*. *Eukaryot Cell* **9**, 215-223, doi:10.1128/EC.00214-09 (2010).

2 Nozaki, T. *et al.* Molecular cloning and characterization of the genes encoding two isoforms of cysteine synthase in the enteric protozoan parasite *Entamoeba histolytica*. *Mol. Biochem. Parasitol.* **97**, 33-44 (1998).

3 Smirnova, E., Shurland, D. L., Ryazantsev, S. N. & van der Bliek, A. M. A human dynamin-related protein controls the distribution of mitochondria. *J. Cell Biol.* **143**, 351-358 (1998).

4 Hamann, L., Buss, H. & Tannich, E. Tetracycline-controlled gene expression in *Entamoeba histolytica*. *Mol. Biochem. Parasitol.* **84**, 83-91 (1997).

5 Ramakrishnan, G., Vines, R. R., Mann, B. J. & Petri, W. A., Jr. A tetracycline-inducible gene expression system in *Entamoeba histolytica*. *Mol. Biochem. Parasitol.* **84**, 93-100 (1997).

6 Nakada-Tsukui, K., Okada, H., Mitra, B. N. & Nozaki, T. Phosphatidylinositol-phosphates mediate cytoskeletal reorganization during phagocytosis via a unique modular protein consisting of RhoGEF/DH and FYVE domains in the parasitic protozoon *Entamoeba histolytica*. *Cell. Microbiol.* **11**, 1471-1491, doi:10.1111/j.1462-5822.2009.01341.x (2009).

7 Saito-Nakano, Y., Yasuda, T., Nakada-Tsukui, K., Leippe, M. & Nozaki, T. Rab5-associated vacuoles play a unique role in phagocytosis of the enteric protozoan parasite *Entamoeba histolytica*. *J. Biol. Chem.* **279**, 49497-49507, doi:10.1074/jbc.M403987200 (2004).

8 Mi-ichi, F., Makiuchi, T., Furukawa, A., Sato, D. & Nozaki, T. Sulfate activation in mitosomes plays an important role in the proliferation of *Entamoeba histolytica*. *PLoS Negl. Trop. Dis.* **5**, e1263, doi:10.1371/journal.pntd.0001263 (2011).

9 Baba, M. Electron microscopy in yeast. *Methods Enzymol.* **451**, 133-149, doi:10.1016/S0076-6879(08)03210-2 (2008).

10 Santos, H. J. *et al.* A novel Mitosomal β-barrel Outer Membrane Protein in *Entamoeba*. *Sci. Rep.* **5**, 8545, doi: 10.1038/srep08545 (2015).

11 De Cadiz, A. E., Jeelani, G., Nakada-Tsukui, K., Caler, E. & Nozaki, T. Transcriptome analysis of encystation in *Entamoeba invadens*. *PLoS One* **8**, e74840, doi:10.1371/journal.pone.0074840 (2013).

12 Ehrenkaufer, G. M. *et al.* The genome and transcriptome of the enteric parasite *Entamoeba invadens*, a model for encystation. *Genome Biol.* **14**, R77, doi:10.1186/gb-2013-14-7-r77 (2013).

13 Roy, M., Reddy, P. H., Iijima, M. & Sesaki, H. Mitochondrial division and fusion in metabolism. *Curr. Opin. Cell Biol.* **33**, 111-118, doi:10.1016/j.ceb.2015.02.001 (2015).

14 Wienke, D. C., Knetsch, M. L., Neuhaus, E. M., Reedy, M. C. & Manstein, D. J. Disruption of a dynamin homologue affects endocytosis, organelle morphology, and cytokinesis in *Dictyostelium discoideum*. *Mol. Biol. Cell* **10**, 225-243 (1999).

15 Schimmel, B. G., Berbusse, G. W. & Naylor, K. Mitochondrial fission and fusion in *Dictyostelium discoideum*: a search for proteins involved in membrane dynamics. *BMC Res. Notes* **5**, 505, doi:10.1186/1756-0500-5-505 (2012).

16 Leger, M. M. *et al.* An ancestral bacterial division system is widespread in eukaryotic mitochondria. *Proc. Natl. Acad. Sci. U. S. A.* **112**, 10239-10246, doi:10.1073/pnas.1421392112 (2015).

17 Sesaki, H. & Jensen, R. E. Ugo1p links the Fzo1p and Mgm1p GTPases for mitochondrial fusion. *J. Biol. Chem.* **279**, 28298-28303, doi:10.1074/jbc.M401363200 (2004).

Makiuchi et al. Supplementary Fig. S1

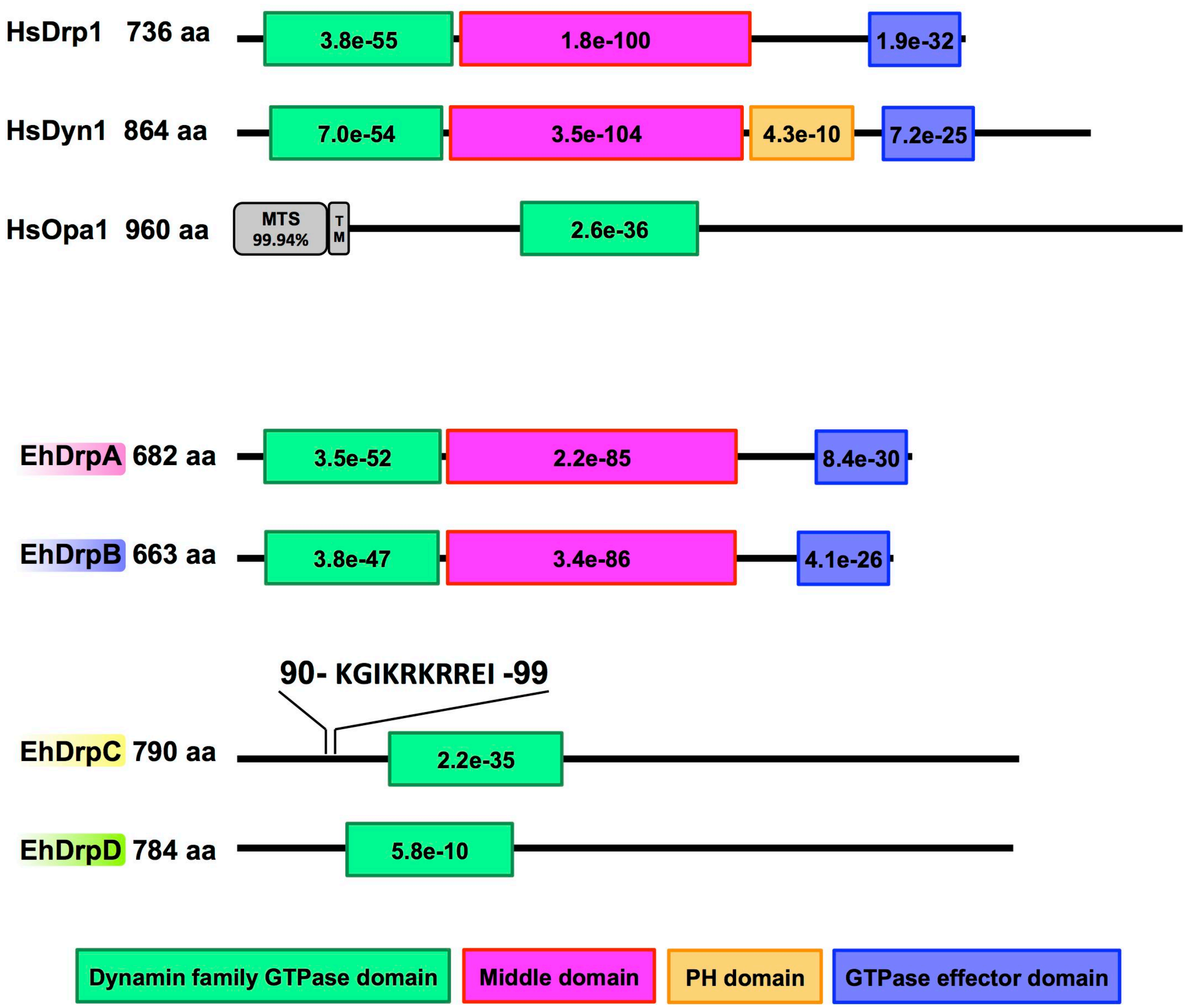

Makiuchi et al. Supplementary Fig. S2

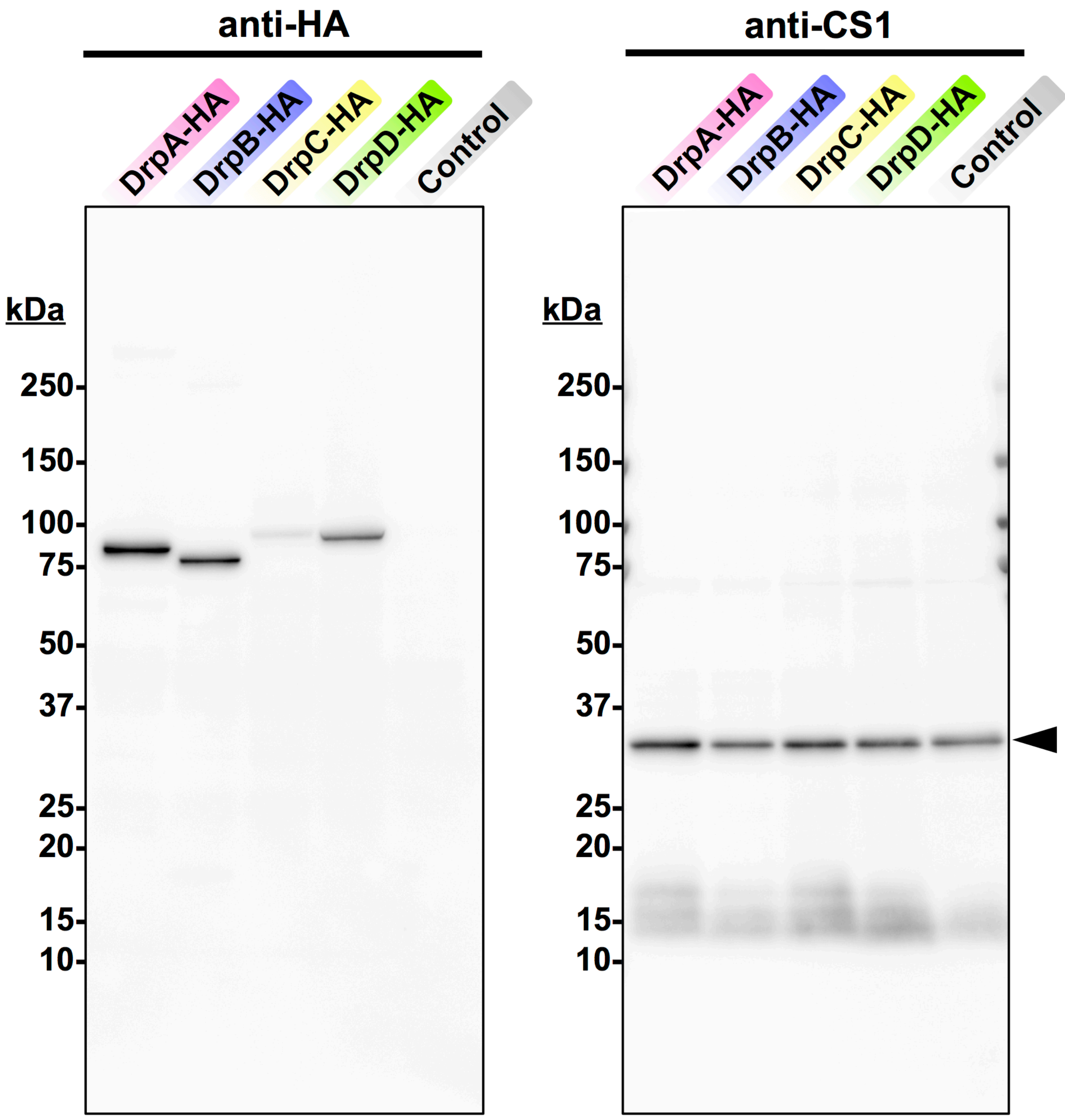

Makiuchi et al. Supplementary Fig. S3

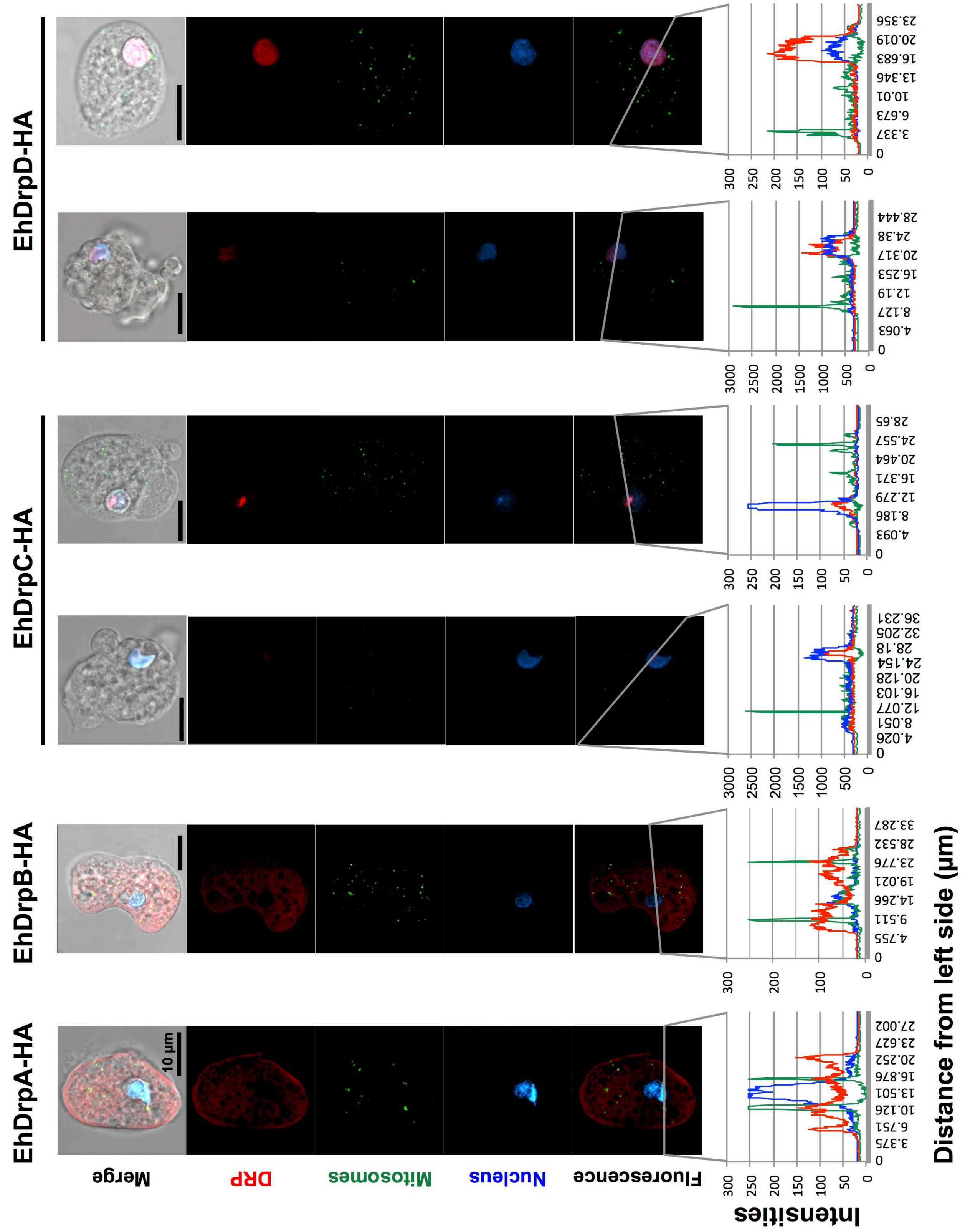

Makiuchi et al. Supplementary Fig. S4

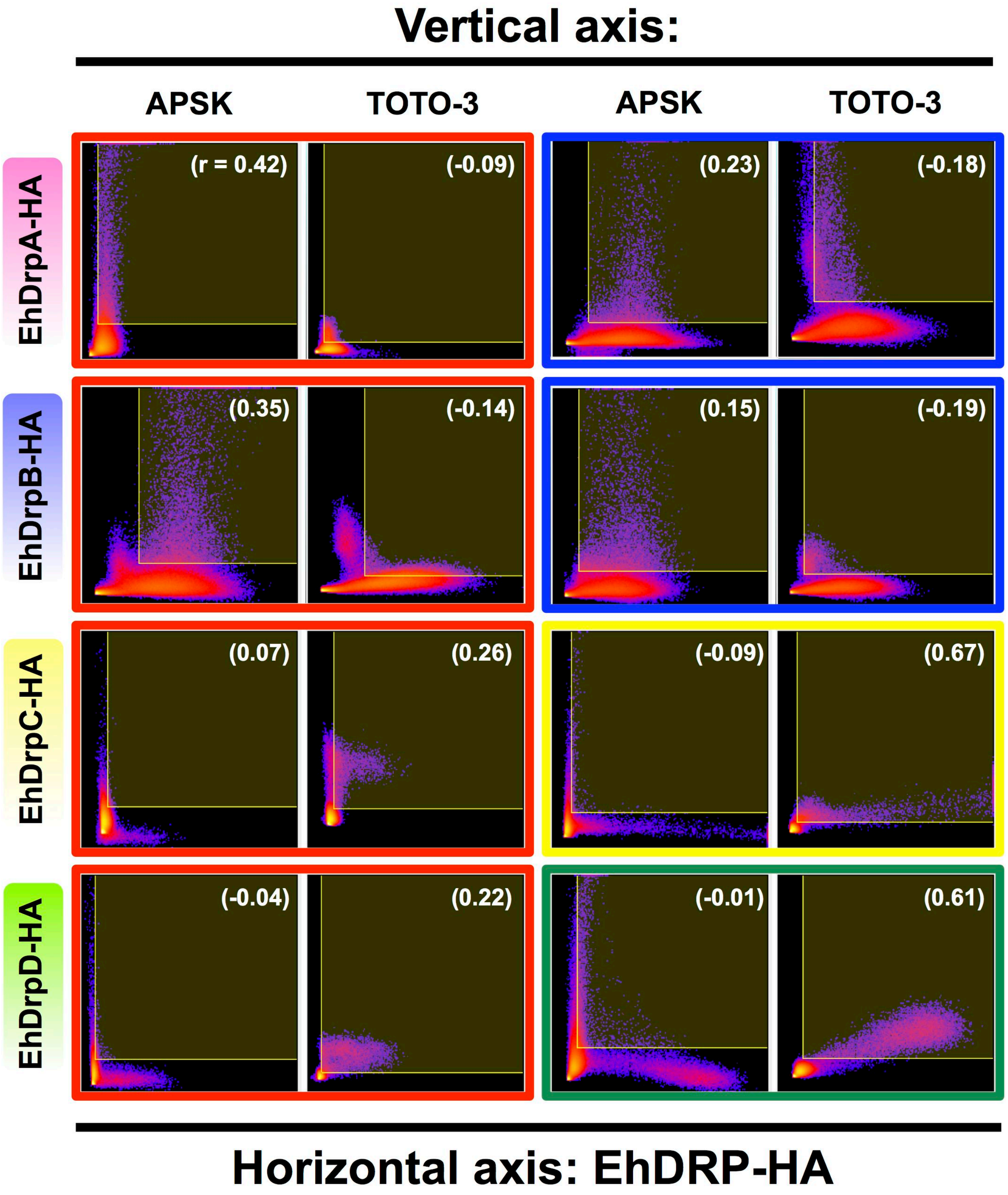

Makiuchi et al. Supplementary Fig. S5

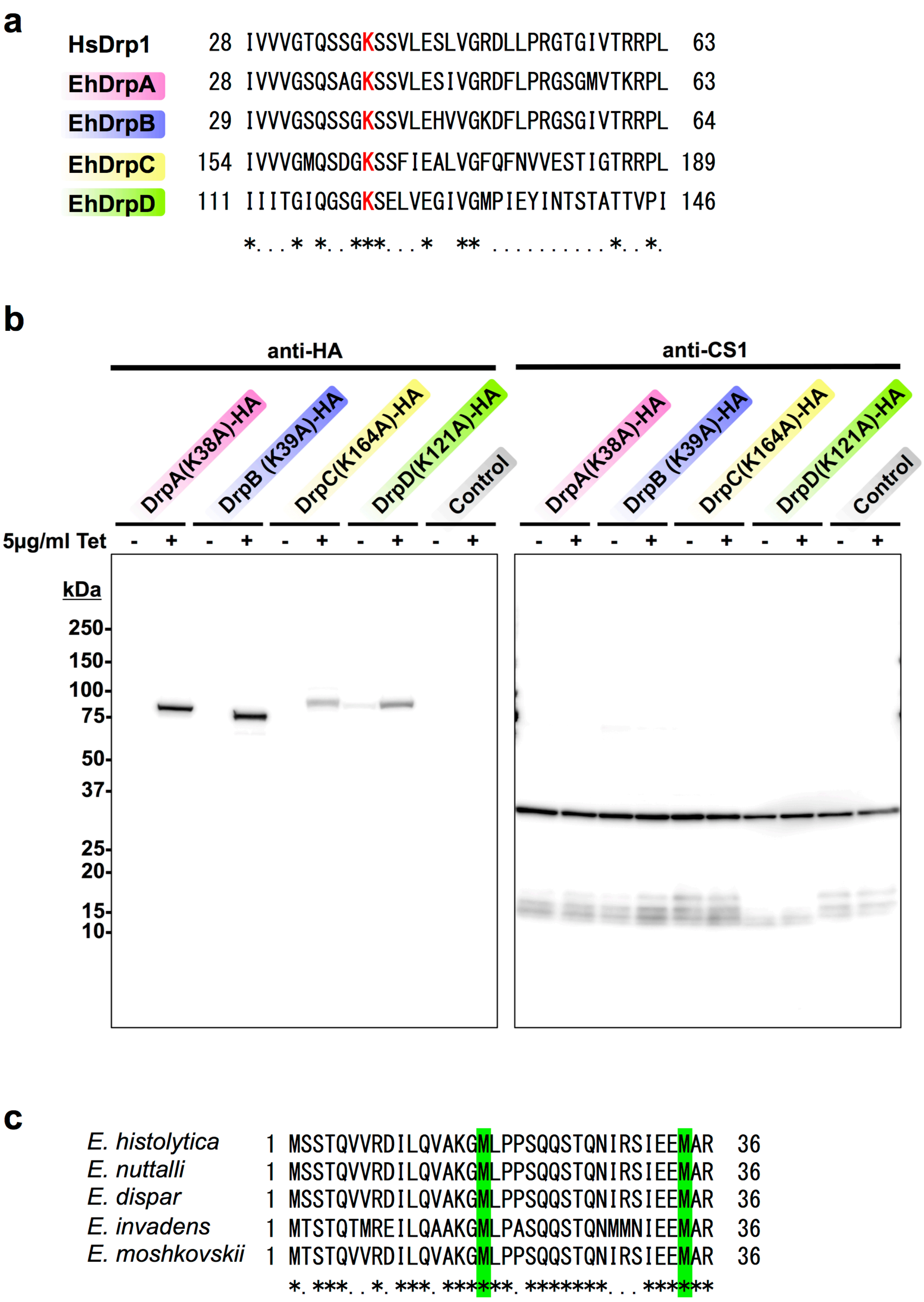

Makiuchi et al. Supplementary Fig. S6

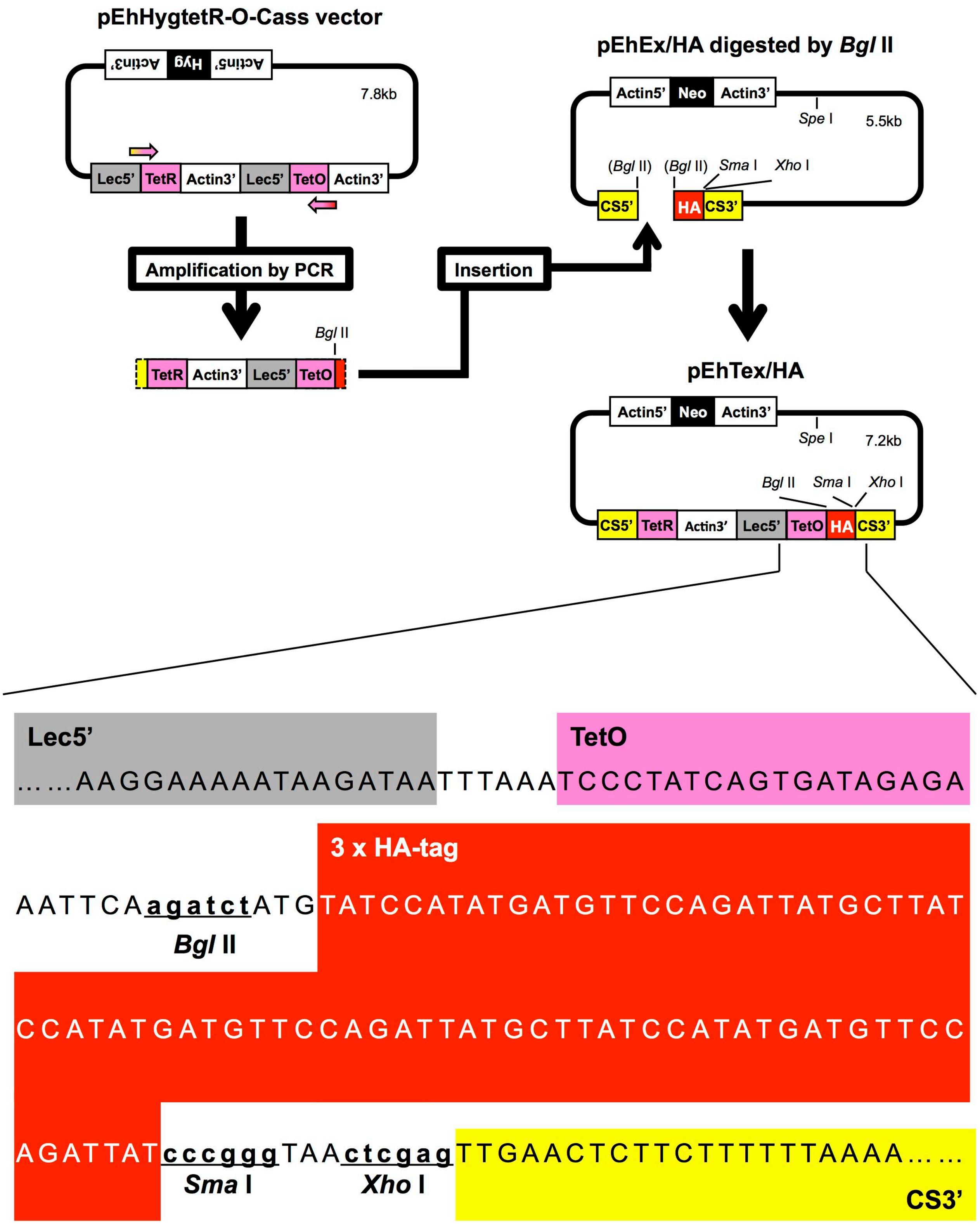

Makiuchi et al. Supplementary Fig. S7

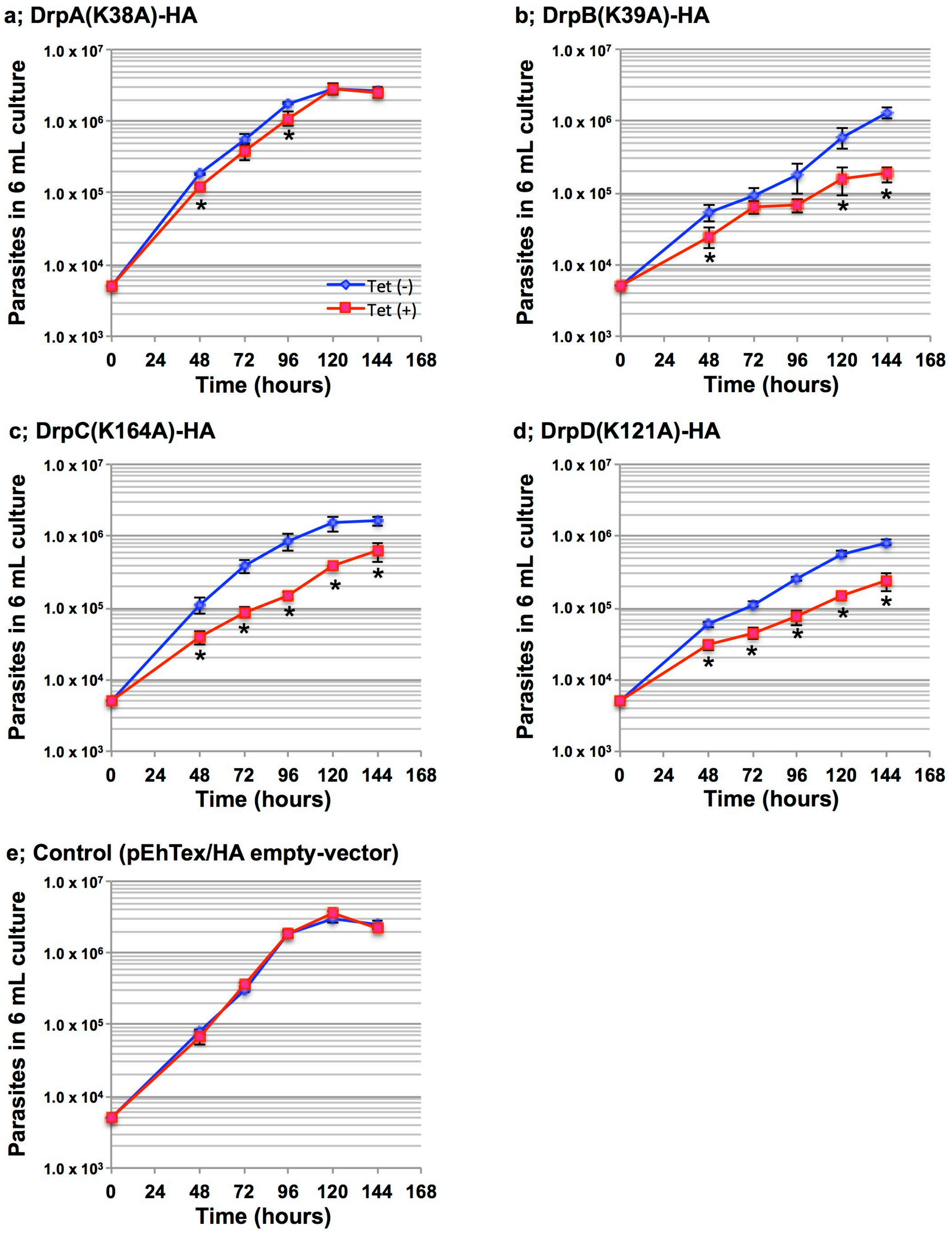

Makiuchi et al. Supplementary Fig. S8

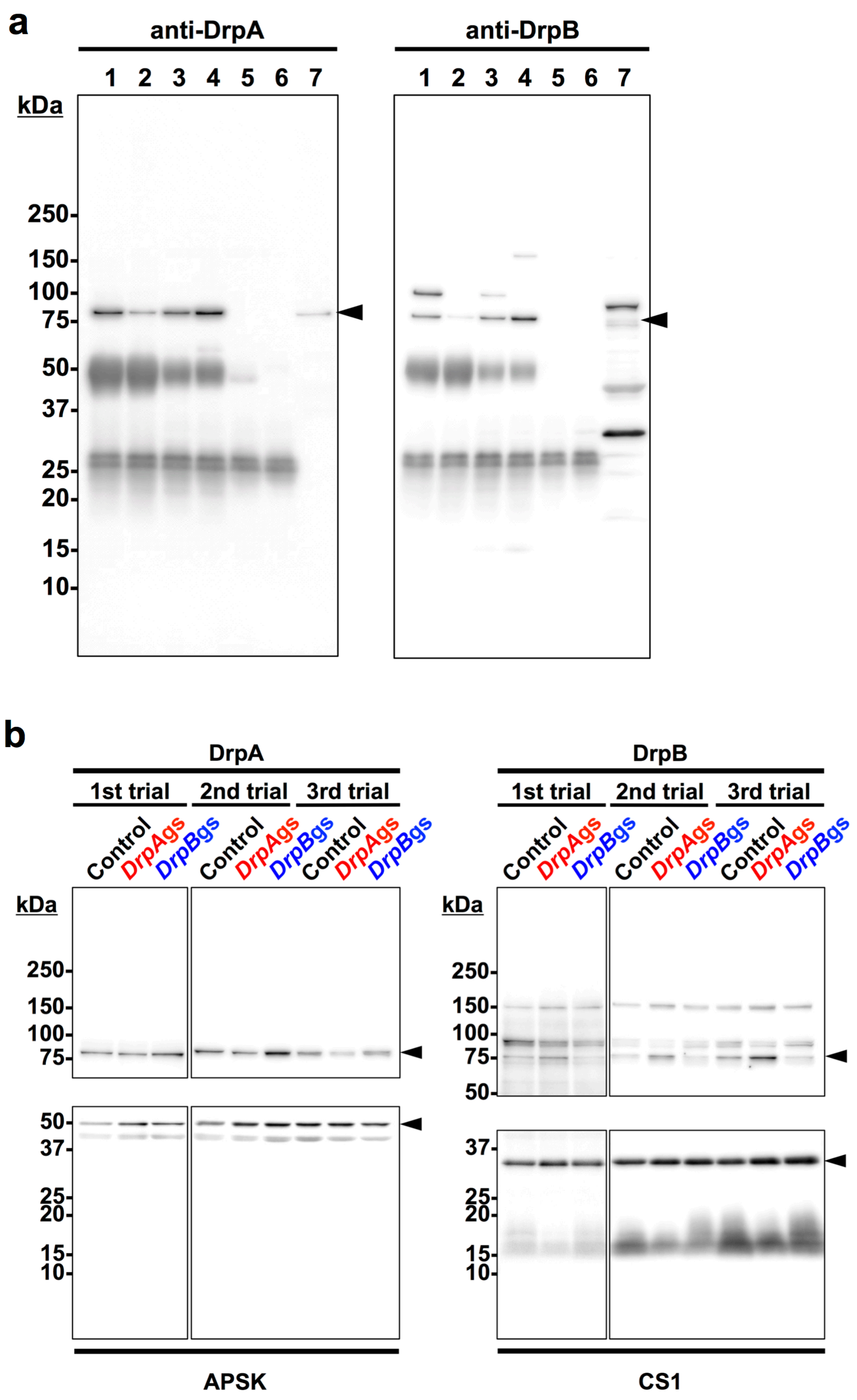

Makiuchi et al. Supplementary Fig. S9

```
EhDrpA     1 MKSLIPVINQLQDVFNTIGV-KGIDLPQIVVVGSQSAGKSSVLESIVGRDFLPRGSGMVT   59
EhDrpB     1 MQRLIPVINSLQDVFTAAGLPNTLPLPQIVVVGSQSSGKSSVLEHVVGKDFLPRGSGIVT   60
             *  ****** *****   *      *********** *******  ** ******** **

EhDrpA    60 KRPLILQLVNLPSTETKEWGEFAHKPGIVYRDFEEIKKEIENETIRLTGTKKTISPVAIR  119
EhDrpB    61 RRPLIVQCVR--SNVAEDYGQFEHTGDRKFTDFGEIRNEITRETER-TCPGRNVSSVPIR  117
              **** * *   *      * * *       ** **  **  ** * *      * * **

EhDrpA   120 LKIYSPYVVDLTLVDLPGLTKISVGSQEKDISNQLKQMVLKFIERPNAIILAVTSANVDL  179
EhDrpB   118 LRIYSSSVVDLTLVDLPGLVKVNINGQTAEMVKNLRDMVYEYASPSNALILAVTAGNIDI  177
             * ***  ************ *     *       *  **       ** *****  * *

EhDrpA   180 ATSDALSIAREVDPDGDRTIGVLTKMDIMDKGTDAMDVLYGRVYPLKLGYIGVLNRSQHD  239
EhDrpB   178 ANSDALQVAKDVDPDGERTIGVLTKLDLEDKGTNSMDVLMGRVYPLKLGYIGVVNRSQQD  237
             * ****  *  ***** ******** *  ****  **** ************* **** *

EhDrpA   240 IDTNVPIKTALTKEKEWFSNHPIYSKIADRLGIPYLTKTLNEILMQHIMKTLPSLRITIT  299
EhDrpB   238 INNGVDVKTSLRHEKEFFENHPVYCSIAERMGTEYMVNRLNVLLLQHIQKCLPGLKQQIN  297
             *   *  ** *  *** * *** *  ** * *  *    **  * *** * ** *   *

EhDrpA   300 EMLNKTKLEYNKFAIEFDQKDVAL-LEKVIEYCTSIQQTISGEKFDIEKHELIGGAKIFD  358
EhDrpB   298 QCYEKARSRYEDIKPD-DENLLSLSLQQIMKFSGSFAAALNGTDTNIHTHEISGGAKIFS  356
                 *    *       *     * *        *      *    *  **  ******

EhDrpA   359 VFENVYRPIIDQLDLIKEISDKDIKTAMKNTEGVNSALFLSQAAFEILVKQQIDKFTDSS  418
EhDrpB   357 VFENNFRPTIDSQDILSGIKDVDILTAIKNASGTRPCLYVPQSAFENLISKQVRNFEGTC  416
             ****  ** **  *    * * ** ** **  *    *   * *** *   *   *

EhDrpA   419 QQCVDKIRKEMSNIFTYVASEVVVRYAKLRDAIIIASDNVLDKNLNKTHEMVKNLIDIEE  478
EhDrpB   417 HNCVDNVYREMKVIVGKIAKDNIEKYDRFREALIQASTEVMNDYMTQTHKMVQDLIDIEA  476
               ***    ** *    *      *   * * * ** *       ** **  *****

EhDrpA   479 SYINTIHPDFDATEIMLNAGINSATPSNEQKPPVVTQAPPKPIPQQPTTKPPKKQSPSKG  538
EhDrpB   477 DYINTSHPDFDTTKVLKEADEAMKTPQDGIDTIVTIDPNNTTNAQQYEAKKPVK-----S  531
              **** ***** *     *     **       *          **   * * *

EhDrpA   539 GFWFWASKASDENEDEDNETQKQTTPSVPVQPEPKVEPIVISATDTKEQRNIKMMRELTR  598
EhDrpB   532 SFFAGQINKNQAKPQQQHVPKESITTSIRVDHTNQREMREINLIRNLCKDYLLIVR---K  588
              *                      * *  *      *   *              *

EhDrpA   599 SYLNIVRKSIEDFVPKAIMHFLINQTCKDL-QKALVEELYKSDKINDLMSESPAITTKRE  657
EhDrpB   589 SIKDLVPKAVIHFLVFKTRDSLQKELIKKLYNEALLQDLL--AENPAIVNERKVVKQNLE  646
             *    * *    *        *     * *   **   *           *        *

EhDrpA   658 MLKKNLEALQKAYNILEGIVTIKVN                                    682
EhDrpB   647 ALKKALDIINQVRDQCF--------                                    663
              *** *
```

Makiuchi et al. Supplementary Fig. S10

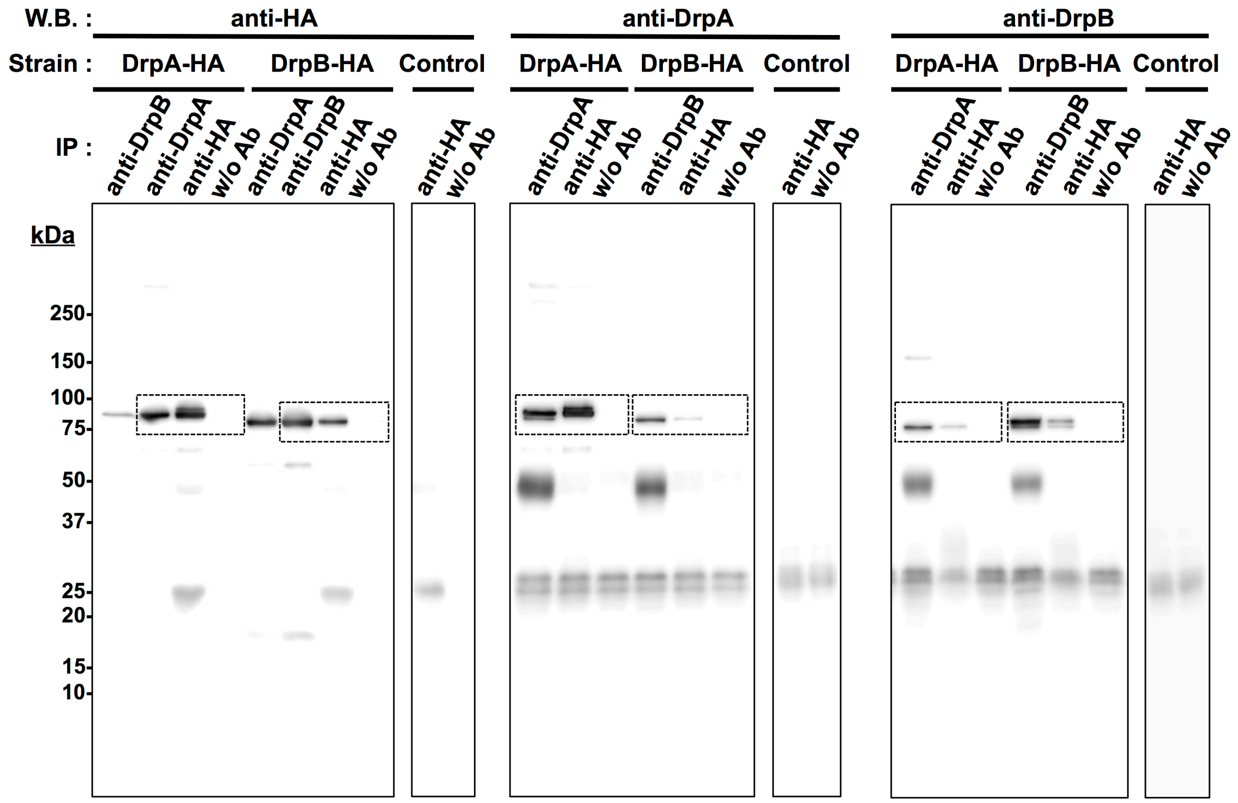

Makiuchi et al. Supplementary Fig. S11

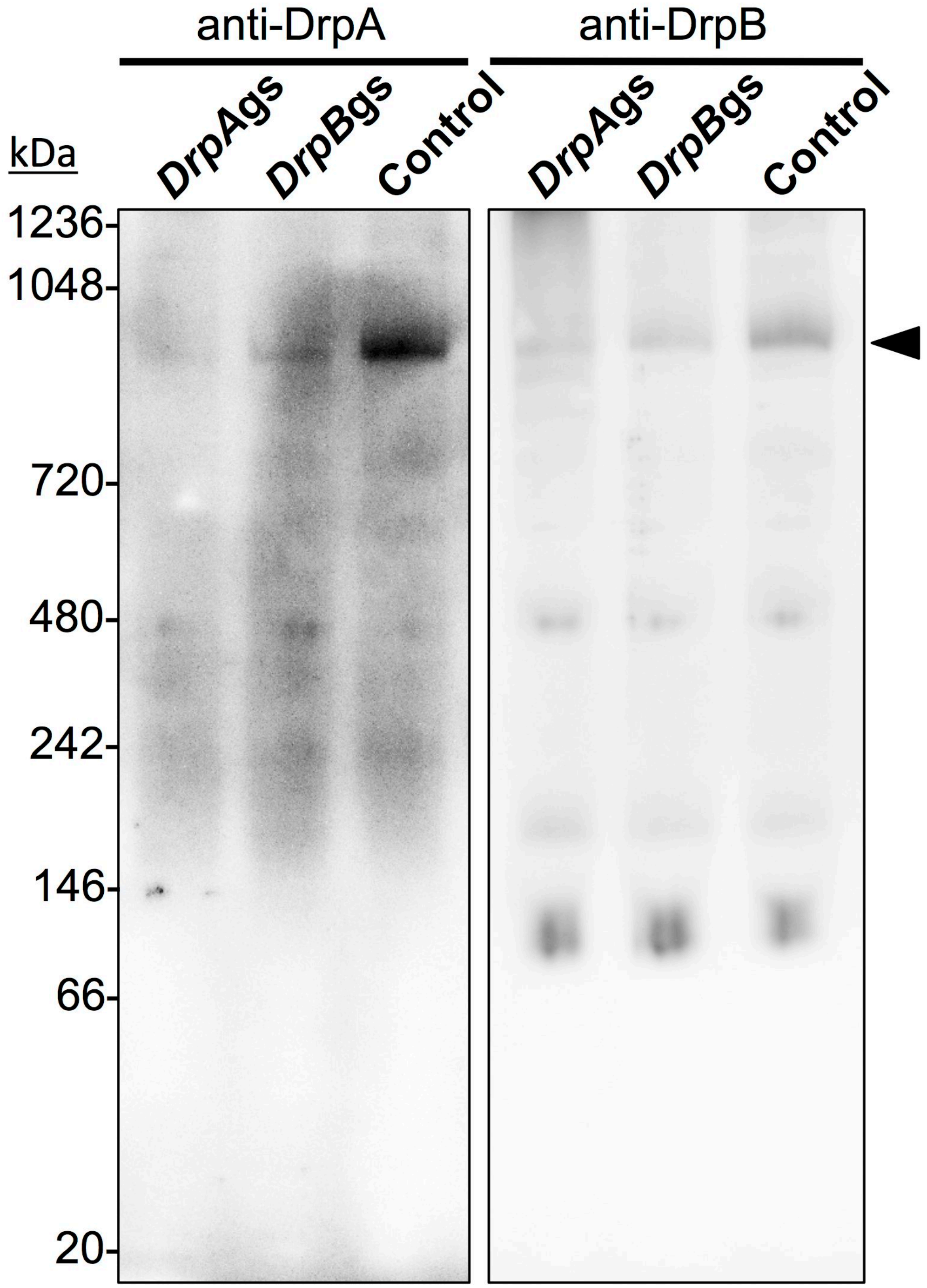

Makiuchi et al. Supplementary Fig. S12

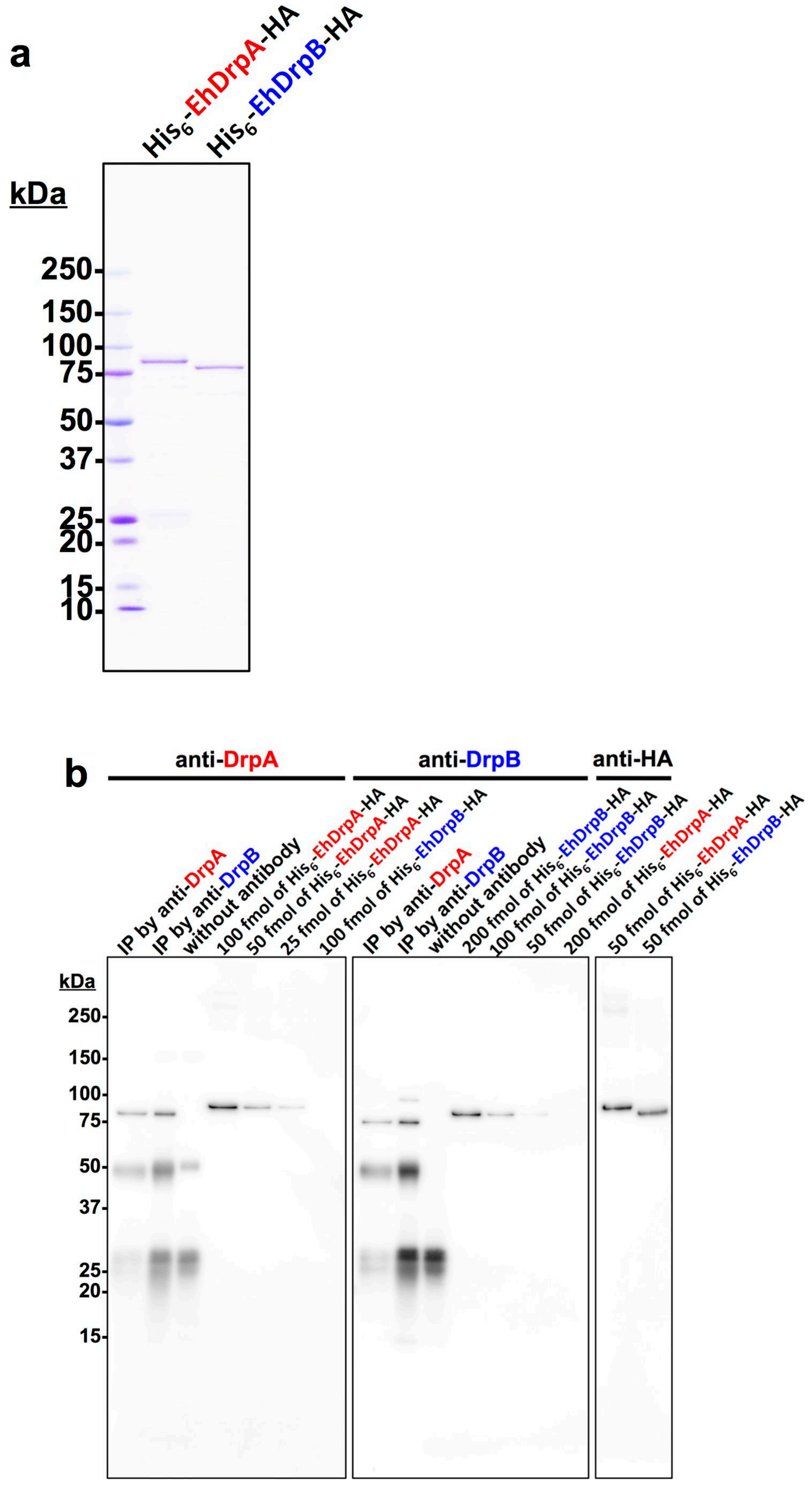

Makiuchi et al. Supplementary Fig. S13

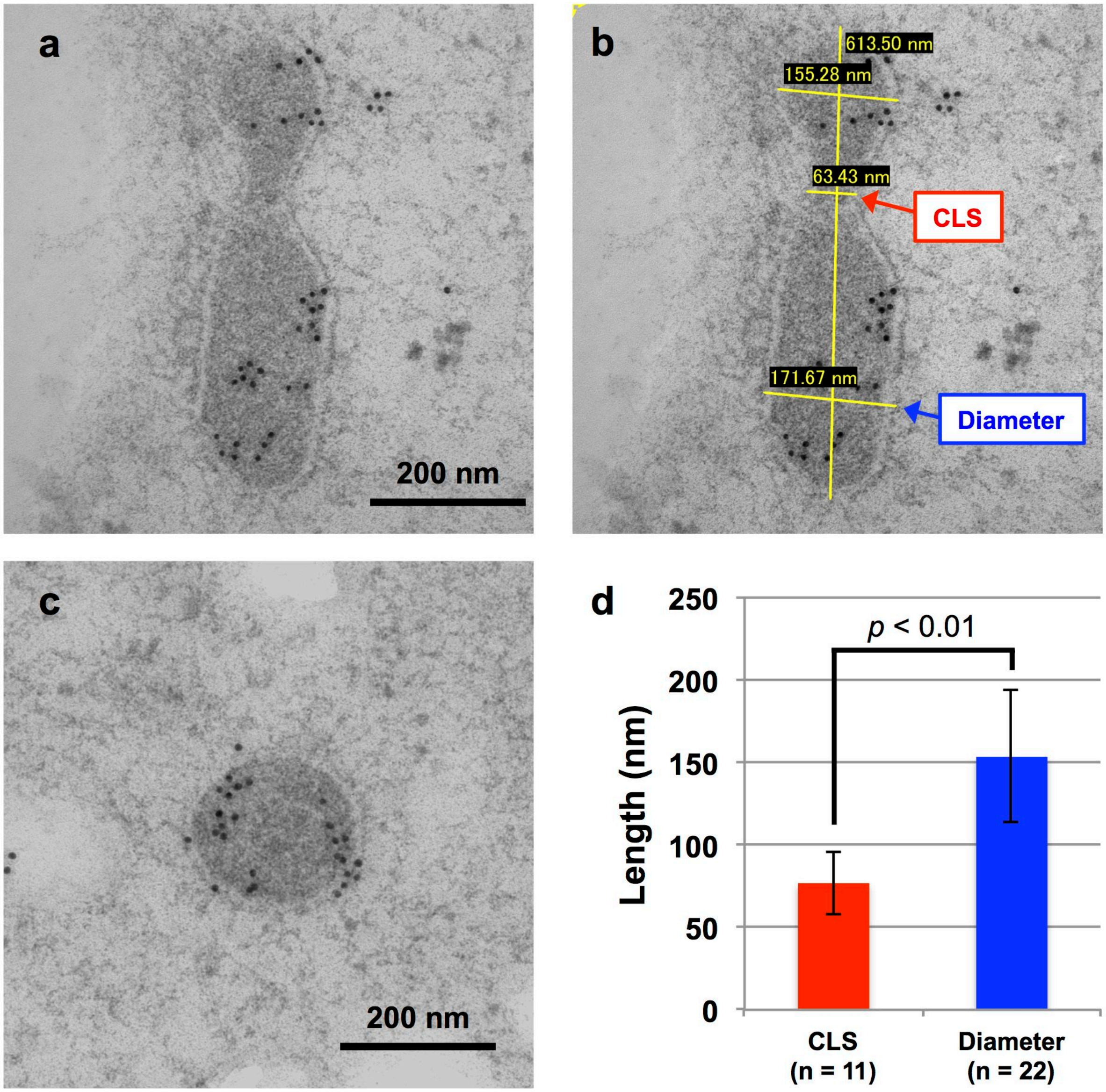

Makiuchi et al. Supplementary Fig. S14

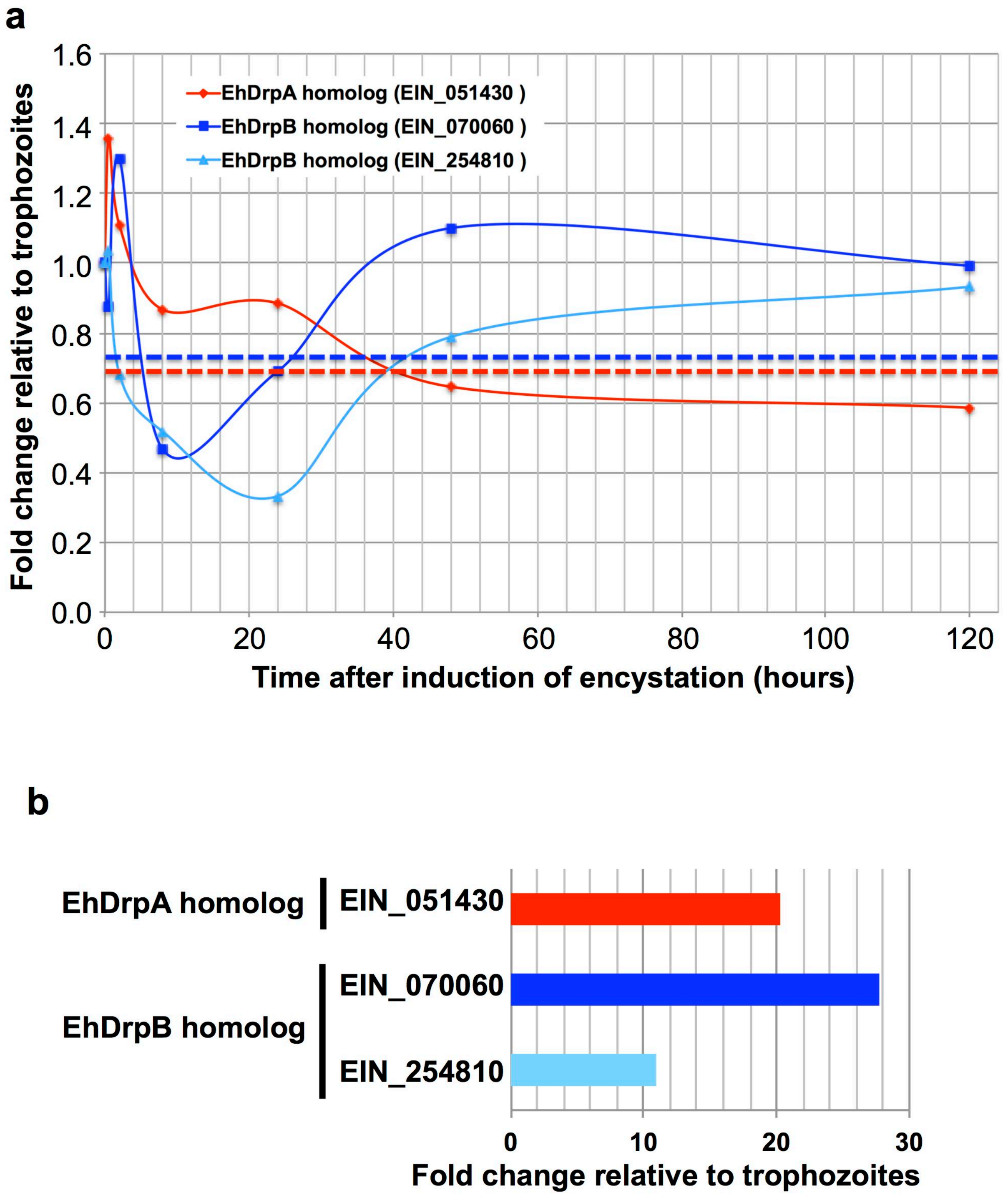

Makiuchi et al. Supplementary Fig. S15

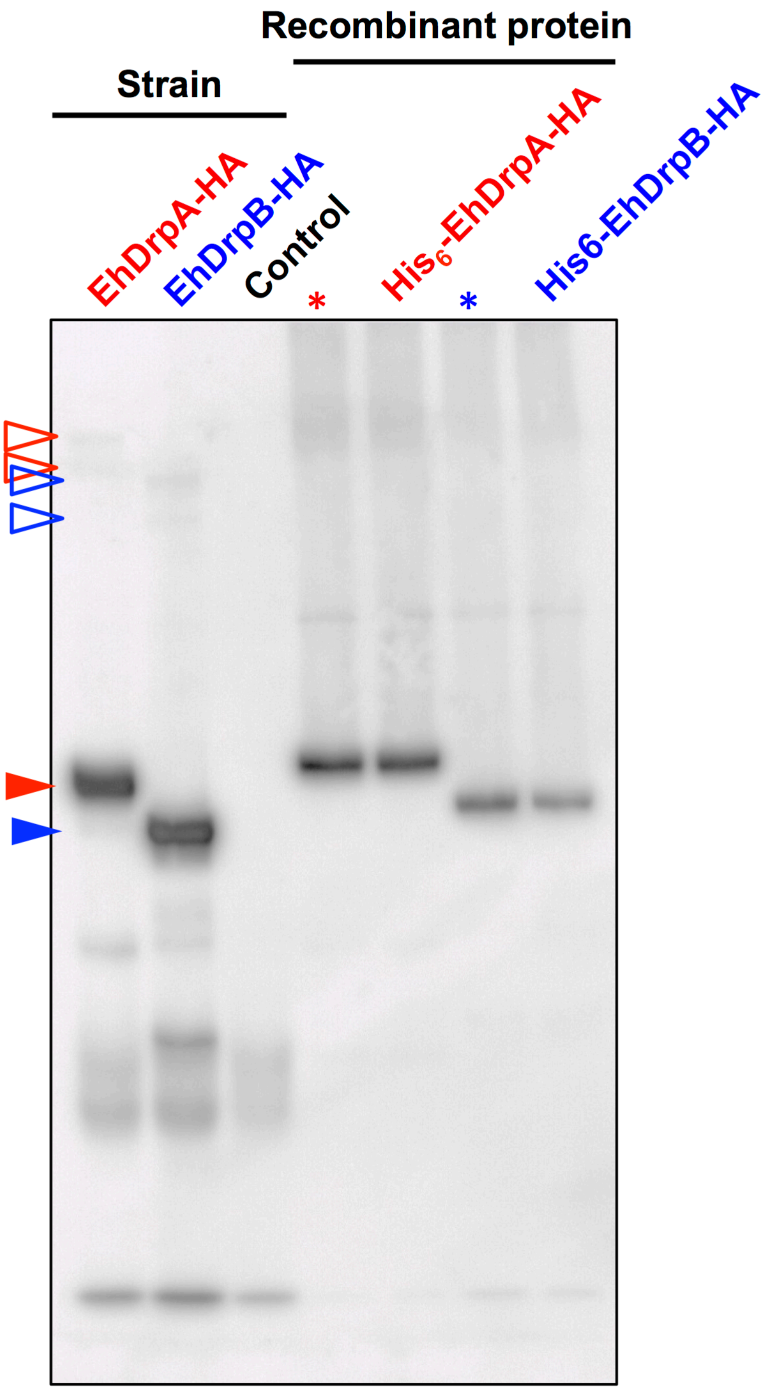

Supplementary Table. S1: List of proteins known to be involved in mitochondrial dynamics

Function	Main components	Mammals	Yeast	*Dictyostelium*	*Entamoeba*	Algae
Outer membrane fission (1)	Dynamin-related protein1 (Drp1)	Drp1	Dnm1	Dynamin A? (Ref. 2 & 3)	Unknown (4 candidates)	Dnm1
DRP receptor protein on outer membarane (1)	Fission protein 1 (Fis1)	Fis1	Fis1	Unknown	Unknown (no homolog)	Fis1
Mammalian DRP adaptoron outer membarane (1)	Mitochondrial fission factor (Mff)	Mff	Unknown	Unknown	Unknown (no homolog)	Unknown
	MiD49 / MiD51	MiD49 / MiD51	Unknown	Unknown	Unknown (no homolog)	Unknown
Yeast Dnm1 adaptor on outer membarane (1)	Mdv1 / Caf4 / Num1 / Mdm36	Unknown	Mdv / Caf4 / Num1 / Mdm36	Unknown	Unknown (no homolog)	Mda1 / - / - / -
Inner membrane fission (4)	FtsZ	Unknown	Unknown	FtsZ	Unknown (no homolog)	FtsZ
Outer membrane fusion (1)	Mitofusin1 / 2 (Mfn1 /Mfn 2)	Mfn1 / Mfn2	Fzo1	Unknown	Unknown (no homolog)	Fzo1
Mediator for the interaction between Fzo1 and Mgm1 (5)	Ugo1	Unknown	Ugo1	Unknown	Unknown (no homolog)	Ugo1
Inner membrane fusion (1)	Optic atrophy 1 (Opa1)	Opa1	Mgm1	Unknown	Unknown (no homolog)	Mgm1

Note:

(1) Roy, M., Reddy, P. H., Iijima, M. & Sesaki, H. Mitochondrial division and fusion in metabolism. *Curr. Opin. Cell Biol.* **33**, 111-118, doi:10.1016/j.ceb.2015.02.001 (2015) (This list is based on this review.). (Ref. No. 13)

(2) Wienke, D. C., Knetsch, M. L., Neuhaus, E. M., Reedy, M. C. & Manstein, D. J. Disruption of a dynamin homologue affects endocytosis, organelle morphology, and cytokinesis in *Dictyostelium discoideum*. *Mol. Biol. Cell* **10**, 225-243 (1999). (Ref. No. 14)

(3) Schimmel, B. G., Berbusse, G. W. & Naylor, K. Mitochondrial fission and fusion in *Dictyostelium discoideum*: a search for proteins involved in membrane dynamics. *BMC Res. Notes* **5**, 505, doi:10.1186/1756-0500-5-505 (2012). (Ref. No. 15)

(4) Leger, M. M. et al. An ancestral bacterial division system is widespread in eukaryotic mitochondria. *Proc. Natl. Acad. Sci. U. S. A.* **112**, 10239-10246, doi:10.1073/pnas.1421392112 (2015). (Ref. No. 16)

(5) Sesaki, H. & Jensen, R. E. Ugo1p links the Fzo1p and Mgm1p GTPases for mitochondrial fusion. *J. Biol. Chem.* **279**, 28298-28303, doi:10.1074/jbc.M401363200 (2004). (Ref. No. 17)

Supplementary Table. S2: List of primers

Purpose	Template for PCR	Sequence	Direction	Mention: Underline	Mention: Bold
Cloning into the *Bgl* II site of pEhEx/HA	pEhHygtetR O Cass	5'-ACACATTAAC**AGATCC**ATGTCTAGATTAGATAAAAG-3'	sense	for In-Fusion® system	for the removal of *Bgl* II restriction site
		5'-ATGGATACAT**AGATCT**TGAATTTCTCTATCACTGATAG-3'	antisense	for In-Fusion® system	*Bgl* II restriction site
Cloning of *EhDrpA* gene into pEhEx/HA digested by *Bgl* II	cDNA	5'-CG**GGATCC**ATGAAAAGTCTTATTCCAGTTATTAAC-3'	sense		*Bam*H I restriction site
		5'-CG**GGATCC**ATTAACTTTGATTGTAACAATTCCTTC-3'	antisense		*Bam*H I restriction site
Cloning of EhDrpB gene intopEhEx/HA digested by *Bgl* II	cDNA	5'-CG**GGATCC**ATGCAAAGATTAATTCCTGTAATTAATAG-3'	sense		*Bam*H I restriction site
		5'-CG**GGATCC**GAAGCATTGATCTCTTACTTGATTAA-3'	antisense		*Bam*H I restriction site
Cloning of EhDrpC gene intopEhEx/HA digested by *Bgl* II	cDNA	5'-CG**GGATCC**ATGAACAACGAACATGAAGAAATC-3'	sense		*Bam*H I restriction site
		5'-CG**GGATCC**ATATTCTACTTCCATAGCGTTCTTA-3'	antisense		*Bam*H I restriction site
Cloning of EhDrpD gene intopEhEx/HA digested by *Bgl* II	cDNA	5'-GTCG**AGATCT**ATGAGCTCAACACAAGTTGTTAG-3'	sense		*Bgl* II restriction site
		5'-GTCG**AGATCT**AAACAATACTCTTTTTTTCATTGGAC-3'	antisense		*Bgl* II restriction site
Cloning of EhDrpA(K38A) gene into pEhTex/HA digested by *Bgl* II and *Xho* I	pEhEx/EhDrpA-HA	5'-GAGAAATTCAAGATCCATGAAAAGTCTTATTC-3'	sense	for In-Fusion® system	
		5'-AGATGA**TGC**ACCAGCACTTTG-3'	antisense	for In-Fusion® system	for the mutation form Lys to Ala
		5'-GCTGGT**GCA**TCATCTGTATTAG-3'	sense	for In-Fusion® system	for the mutation form Lys to Ala
		5'-GAAGAGTTCAACTCGAGTTACC-3'	antisense	for In-Fusion® system	
Cloning of EhDrpB(K39A) gene into pEhTex/HA digested by *Bgl* II and *Xho* I	pEhEx/EhDrpB-HA	5'-GAGAAATTCAAGATCCATGCAAAGATTAATTC-3'	sense	for In-Fusion® system	
		5'-TGAAGA**TGC**TCCAGATGACTG-3'	antisense	for In-Fusion® system	for the mutation form Lys to Ala
		5'-TCTGGA**GCA**TCTTCAGTATTAG-3'	sense	for In-Fusion® system	for the mutation form Lys to Ala
		5'-GAAGAGTTCAACTCGAGTTACC-3'	antisense	for In-Fusion® system	
Cloning of EhDrpC(K164A) gene into pEhTex/HA digested by *Bgl* II and *Xho* I	pEhEx/EhDrpC-HA	5'-GAGAAATTCAAGATCCATGAACAACGAACATG-3'	sense	for In-Fusion® system	
		5'-ACTACT**TGC**CCCATCACTTTGC-3'	antisense	for In-Fusion® system	for the mutation form Lys to Ala
		5'-GATGGG**GCA**AGTAGTTTTATTG-3'	sense	for In-Fusion® system	for the mutation form Lys to Ala
		5'-GAAGAGTTCAACTCGAGTTACC-3'	antisense	for In-Fusion® system	
Cloning of EhDrpD(K121A) gene into pEhTex/HA digested by *Bgl* II and *Xho* I	pEhEx/EhDrpD-HA	5'-GAGAAATTCAAGATCTATGAGCTCAACACAAG-3'	sense	for In-Fusion® system	
		5'-TTCTGA**TGC**ACCAGAGCCTTG-3'		for In-Fusion® system	for the mutation form Lys to Ala
		5'-TCTGGT**GCA**TCAGAATTAGTTG-3'		for In-Fusion® system	for the mutation form Lys to Ala
		5'-GAAGAGTTCAACTCGAGTTACC-3'	antisense	for In-Fusion® system	
Making *DrpA*gs vector	pEhEx/EhDrpA-HA	5'-CC**AGGCCT**ATGAAAAGTCTTATTCCAGTTA-3'	sense		*Stu* I restriction site
		5'-GTCG**GAGCTC**GAATTGCAACTGGTGAAATTG-3'	antisense		*Sac* I restriction site
Making *DrpB*gs vector	pEhEx/EhDrpB-HA	5'-CC**AGGCCT**ATGCAAAGATTAATTCCTGTAAT-3'	sense		*Stu* I restriction site
		5'-GTCG**GAGCTC**TACCAGGACAAGTACGTTC-3'	antisense		*Sac* I restriction site
Making vector for the expression of His-EhDrpA-HA	pEhEx/EhDrpA-HA	5'-GGC**GAGCTC**GAAAACCTGTATTTTCAGGGAAAAAGTCTTATTCCAG-3'	sense	TEV protease cleavage site	*Sac* I restriction site
		5'-GG**GGTACC**AACTCGAGTTACCC-3'	antisense		*Kpn* I restriction site
Making vector for the expression of His-EhDrpA-HA	pEhEx/EhDrpB-HA	5'-GGC**GAGCTC**GAAAACCTGTATTTTCAGGGACAAAGATTAATTCCTG-3'	sense	TEV protease cleavage site	*Sac* I restriction site
		5'-GGGGTACCAACTCGAGTTACCC-3'	antisense		*Kpn* I restriction site
